# New Osmocene and Ruthenocene Phases Reveal the Common Conformational Behavior Regulated by Anagostic Bonds in Prototypical Metallocenes

**DOI:** 10.1021/acs.jpclett.5c00686

**Published:** 2025-06-03

**Authors:** Ida Moszczyńska, Marek Szafrański, Andrzej Katrusiak

**Affiliations:** † Department of Materials Chemistry, Faculty of Chemistry, 467899 Adam Mickiewicz University, Uniwersytetu Poznańskiego 8, 61-614 Poznań, Poland; ‡ Faculty of Physics, Adam Mickiewicz University, Uniwersytetu Poznańskiego 2, 61-614 Poznań, Poland

## Abstract

Ruthenocene and osmocene, under normal conditions isostructural to low-temperature ferrocene phase III, have been regarded as prototypical metallocenes exclusively present in the energetically favored eclipsed conformation. This strong preference contrasts with ferrocene, which exhibits the staggered, rotated, eclipsed, disordered, and modulated conformations in its five polymorphic forms. Here we show that ruthenocene at 394.0 K and osmocene at 421.5 K transform to new higher-symmetry isostructural phases, where the cyclopentadienyl rings become disordered in two modes: seesaw tilts hinged on the metal cation and rotations about the molecular pseudo-*C*_5_ axis. The transitions entropy change and the Fourier transformations of the diffraction data indicate the hindered rotations, with molecules dynamically disordered between the staggered and eclipsed conformations, whereas in the final structural models the electron density distribution around the rings, calculated from the atomic displacement parameters of refined atomic sites, is continuous. For the prototypical metallocenes, a common pattern of transformations leading to disordered conformations has been connected with intramolecular anagostic bonds CH···*M* (*M* = Fe, Ni, Ru, Os). Their strength correlates with the critical temperatures of phase transitions when the anagostic bonds are broken.

## Graphical abstract

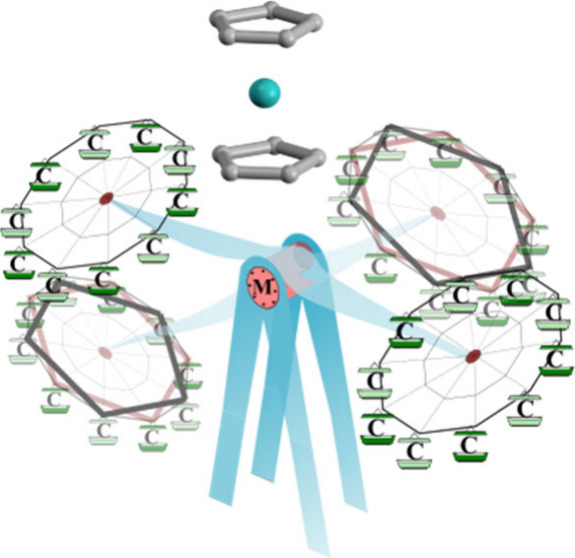

Prototypical metallocenes *M*Cp_2_, where *M* is a metal dication and Cp denotes the cyclopentadienyl [C_5_H_5_]^−^ anion, are textbook examples of conformationally dependent solid-state phases.^1–9^ Both gas-phase electron diffraction^10,11^ and theoretical calculations^12,13^ consistently indicate that the eclipsed conformer of ferrocene (FeCp_2_) is 3.8 ± 1.3 kJ mol^–1^ more stable than the staggered one. Ferrocene is a stunning example of different crystal phases depending on the molecular conformation. The conformation, i.e., the position of one Cp ring (Cp1) relative to the other (Cp2), is measured by torsion angle τ (C–Cp1 centroid–Cp2 centroid–C′) between the closest C atoms of the opposite Cp rings around the (pseudo)-*D*_5_ axis. Ferrocene in its ambient phase I initially was associated only with the staggered conformation (τ = 36°),^14^ but the disorder of the Cp rings^15,16^ implies an entanglement of populations of other conformers,^17^ too. A high pressure of 3.24 GPa is needed to order the Cp rings exclusively in the staggered conformation.^7,18–20^ At ambient pressure (0.1 MPa), below 172.8 K, ferrocene transforms to the conformationally modulated disordered phase I″;^21^ below 163.5 K, in phase II, the molecules order in independent conformers rotated right and left (τ = ±8° and ±9°),^1^ and in subsequent phase III the molecules assume the eclipsed conformation (τ = 0°).^2^ The low-temperature ferrocene phase III is isostructural with ambient-pressure phase α of ruthenocene^22^ and osmocene.^4^ Recently, we obtained new structurally different high-pressure phase β of ruthenocene (space group *Pcmb*) and high-pressure phase β of osmocene (space group *Pcab*), but their conformers remained eclipsed.^23,24^ This exceptional stability of ruthenocene (RuCp_2_)^22,23^ and osmocene (OsCp_2_)^4,24^ in the eclipsed conformation was puzzling when taking into account the similar molecular dimensions (distance *M*–Cp at ambient conditions in ferrocene is 1.651 Å,^18^ that in ruthenocene is 1.812 Å, and that in osmocene is 1.815 Å) as those of ferrocene, the same configuration of 18 valence electrons as well as the similar potential energy favoring the eclipsed conformers^10–12,25–27^ by ca. 4 kJ mol^–1^, and when confronted with the partly or fully built staggered conformers, ferrocene phases I, I′ and I″. Nickelocene,^28,29^ vanadocene,^30^ chromocene,^31^ cobaltocene,^28,32^ and magnesocene^33^ were reported only in the disordered or staggered conformations. It is even more puzzling that ferrocene has been so far the only known metallocene crystal that topochemically transforms between staggered and eclipsed phases.^2,9,34,35^ Recently, we revealed the systematic increase of the H-acceptor capability of the central *M* ion in the series from ferrocene to ruthenocene and osmocene molecules.^24^ The anagostic CH···Ru bonds between molecules, postulated by Borissova et al.^36^ for ruthenocene phase α, become clearly more pronounced in high-pressure phase β, where they strongly stabilize the eclipsed conformer.^23,24^ Likewise, short CH···Os bonds stabilize the eclipsed conformers of osmocene in its high-pressure phase β. The absence of short CH···*M* contacts in ferrocene coincides with the domination of its *p*-*T* diagram by the disordered and staggered phases I, I′ and I″. Here we report the high-temperature breaking of the CH···Ru bonds in ruthenocene and the CH···Os bonds in osmocene, which induces the conformational transitions in these compounds. These observations provide the consistent landscape common for all prototypic metallocenes and their thermodynamic properties, at last.

Our calorimetric measurements show that the ruthenocene and osmocene crystals undergo high-temperature phase transitions at 394.0 and 421.5 K, respectively (Figure 1). The second-order character of these transitions is evident from the shape of the thermal anomalies and the lack of temperature hysteresis between the cooling and heating runs (cf. Figures S1 and S2 in the Supporting Information). This is also confirmed by the continuous change in entropy over a wide temperature range. The total gain in entropy accompanying the transition in ruthenocene amounts to Δ*S* = 11.5(6) J mol^–1^ K^–1^. This value accounts for the configurational-entropy change, Δ*S* = *R*ln­(*N*_1_/*N*_2_), where *N*_1_/*N*_2_ = 4 is the ratio of configurations numbers in the high-temperature (*N*_1_) and low-temperature (*N*_2_) phases and *R* is the gas constant. For osmocene, the transition entropy is also high, Δ*S* = 9.9(5) J mol^–1^ K^–1^, which corresponds to Δ*S* = *R*ln 3.3. These entropy changes clearly indicate the order–disorder mechanism of the transitions, where the high-temperature phases exhibit a 4-fold increase in the number of disorder sites. Furthermore, the stretch of observed thermal anomalies indicates that the disorder activation starts around 150 K below the critical temperatures (*T*_*c*_ – 150 K), as indicated in Figure 1 (cf. Figures S1–S4 in the Supporting Information).

**Figure 1:**
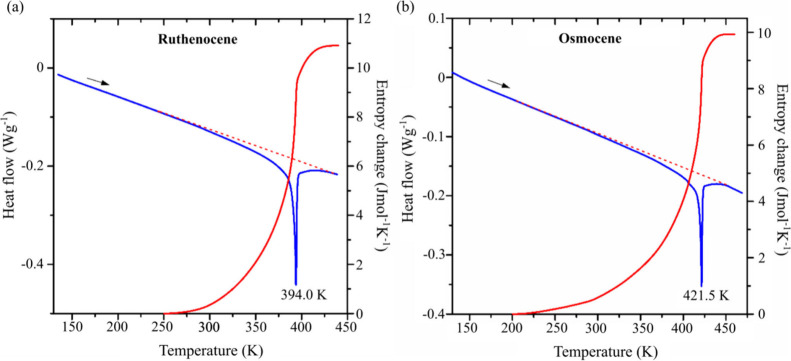
DSC heating runs (blue) and entropy changes (red) for (a) ruthenocene and (b) osmocene. The dashed lines indicate the baselines.

The single-crystal X-ray diffraction (SCXRD) structural studies of the new γ phases of ruthenocene and osmocene were challenging due to the strong sublimation of these compounds above 350 K. To prevent the sublimation, we fixed the samples between cotton fibers gently pushed by a glass rod nearly matching the inner diameter of the capillary; we additionally filled the space between the rod and capillary with Distal (a two-component polymer glue). The capillaries were sealed by melting their ends in a microtorch: one capillary end was sealed first, then the cotton–sample–cotton–glass rod were inserted, and then the other end was melted together with the glass rod end. In the temperature range close to the *T*_c_ and in the γ phase, these protections of the sample crystal were sufficient to collect one full data set (during 30 min) and to start the subsequent one: after 5–10 min, the sample crystal started to move due to its sublimation. During one 30 min data collection, the intensity of control reflections dropped by about 90%; the reflection intensities accounted for this sublimation effect (cf. Figure S5). Despite these experimental difficulties, the structures were solved straightforwardly by direct methods in Shelxs,^37^ and in the Fourier maps the electron density peaks clearly indicated the carbon atoms of the Cp ring disordered in four sites, two due to the seesaw movement of molecules about the [*y*]-axis and another two due to the rotation of the Cp rings by 36° about the pseudo-*C*_5_ molecular axis. These clear locations of 4-fold disordered Cp rings were consistent for the γ phases of ruthenocene and osmocene, and their 4-fold disorder indicated by the entropy gains, associated with the transitions between the α and γ phases, were close to *R*ln 4 (see the calorimetric results presented above). All this information consistently confirmed the disordering mode, where the Cp ring jumps between two sites 36° apart about the pseudo-*C*_5_ axis and between two sites one inclined by 60° to the other in the seesaw mode about the [*z*]-axis. The new γ phases of ruthenocene and osmocene, determined by SCXRD (cf. detailed information about the measurements and crystals are available in Experimental Section, in the Supporting Information, and in Table S1) at 400 K for RuCp_2_ and 427 K for OsCp_2_, are isostructural (Table 1). The group–subgroup symmetry relations between phases γ and α are consistent with the continuous character of the transitions. The unit-cell parameters and thermal expansions of RuCp_2_ and OsCp_2_ phases α and γ are similar (Figure 2). The thermal expansion of parameters *a* and *b* clearly increases when approaching *T*_c_, whereas the thermal expansion of parameter *c* remains nearly constant. Above the transition temperatures, the *c* parameters display a strong negative thermal expansion, also observed for parameters *a* and *b* of ruthenocene and parameter *b* of osmocene.

**Table 1:** Selected Crystal Data of Ruthenocene and Osmocene Phases α and γ^a^

	**RuCp**_**2**_		**OsCp**_**2**_	
Phase	α	γ	α	γ
Temperature	290 K	400 K	293 K	427 K
Space group	*Pnma*	*Fmmm*	*Pnma*	*Fmmm*
*a* (Å)	7.1097(1)	7.2161(5)	7.0848(3)	7.1956(4)
*b* (Å)	8.9752(2)	9.2195(6)	8.9122(5)	9.1560(5)
*c* (Å)	12.7934(3)	12.7724(6)	12.7883(7)	12.7736(7)
*V* (Å^3^)	816.36(3)	849.73(9)	807.47(7)	841.56(8)
*Z*	4	4	4	4
Conformation	eclipsed	staggered 50%, eclipsed 50%	eclipsed	staggered 50%, eclipsed 50%

a See Table S1 in the Supporting Information.

**Figure 2:**
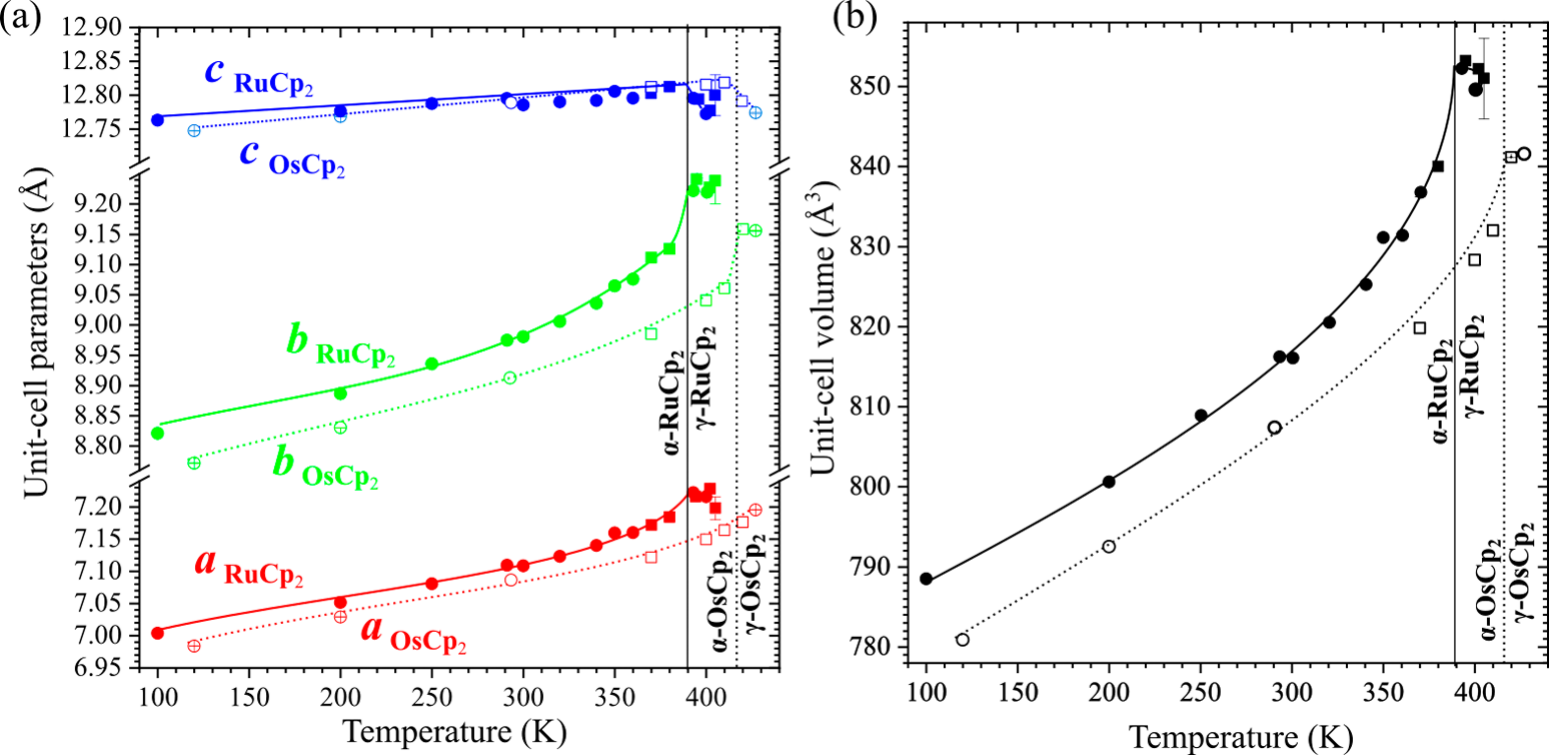
Thermal expansion of (a) unit-cell parameters of RuCp_2_ (full symbols, solid lines) and OsCp_2_ (open symbols, dotted lines) and (b) volume. Vertical solid and dotted lines mark the phase transitions. Squares and circles indicate the PXRD and SCXRD data, respectively. All estimated standard deviations (ESDs) are smaller than the plotted symbols.

This thermal expansion reflects structural transformations. The Ru and Os atoms remain at the *D*_2*h*_-symmetric special position, at the intersection of three mirror planes (Figures 3 and 4). At *T*_*c*_, the RuCp_2_ and OsCp_2_ molecules become disordered in an unprecedented manner for all metallocenes. This disorder can be decomposed into two modes: (i) the seesaw motion of the molecules about the [*y*]-direction measured by angle α_[*z*]_ between the molecular pseudo-*C*_5_ axis and the [*z*]-direction, angle α_[*z*]_, is close to ±30° (cf. Figures 3, 4, and S6) and (ii) the Cp-ring rotations by angle φ = 36° about the molecular pseudo-*C*_5_ axis (Figures 3 and 4). The seesaw disorder (i) leads to two half-occupied sites of each Cp ring, and (ii) each of these time-averaged half Cp rings is further divided into two 0.25-occupied sites due to the pseudo-*C*_10_ rotations mode. Two Cp1-ring sites A and B (Figure S7) are symmetry-independent, and they can assume different site occupation factors SOF_A_ and SOF_B_ (= 0.5 – SOF_A_). Our refinements of both structures γ-RuCp_2_ and γ-OsCp_2_ yielded SOF_A_ = 0.25(3) and 0.25(2), respectively; hence, we fixed the SOF values at 0.25 in the final refinements. Thus, each Cp ring becomes disordered in four equally 0.25-occupied positions. The connected symmetry change is consistent with the number of 4 configurations derived from the calorimetric measurements.

**Figure 3:**
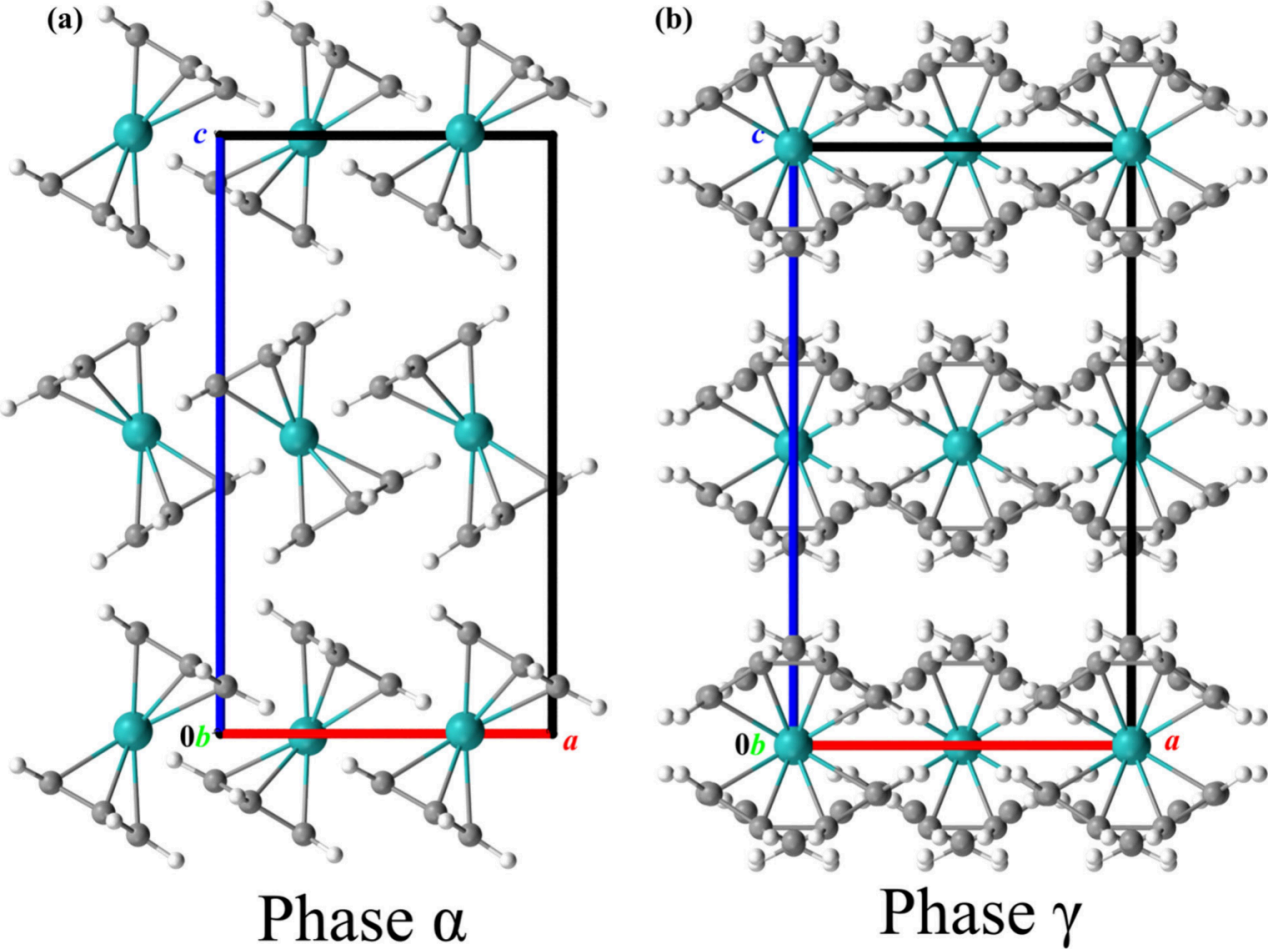
Crystal structures of RuCp_2_ (a) in phase α at 293 K and (b) in phase γ at 395 K.

**Figure 4:**
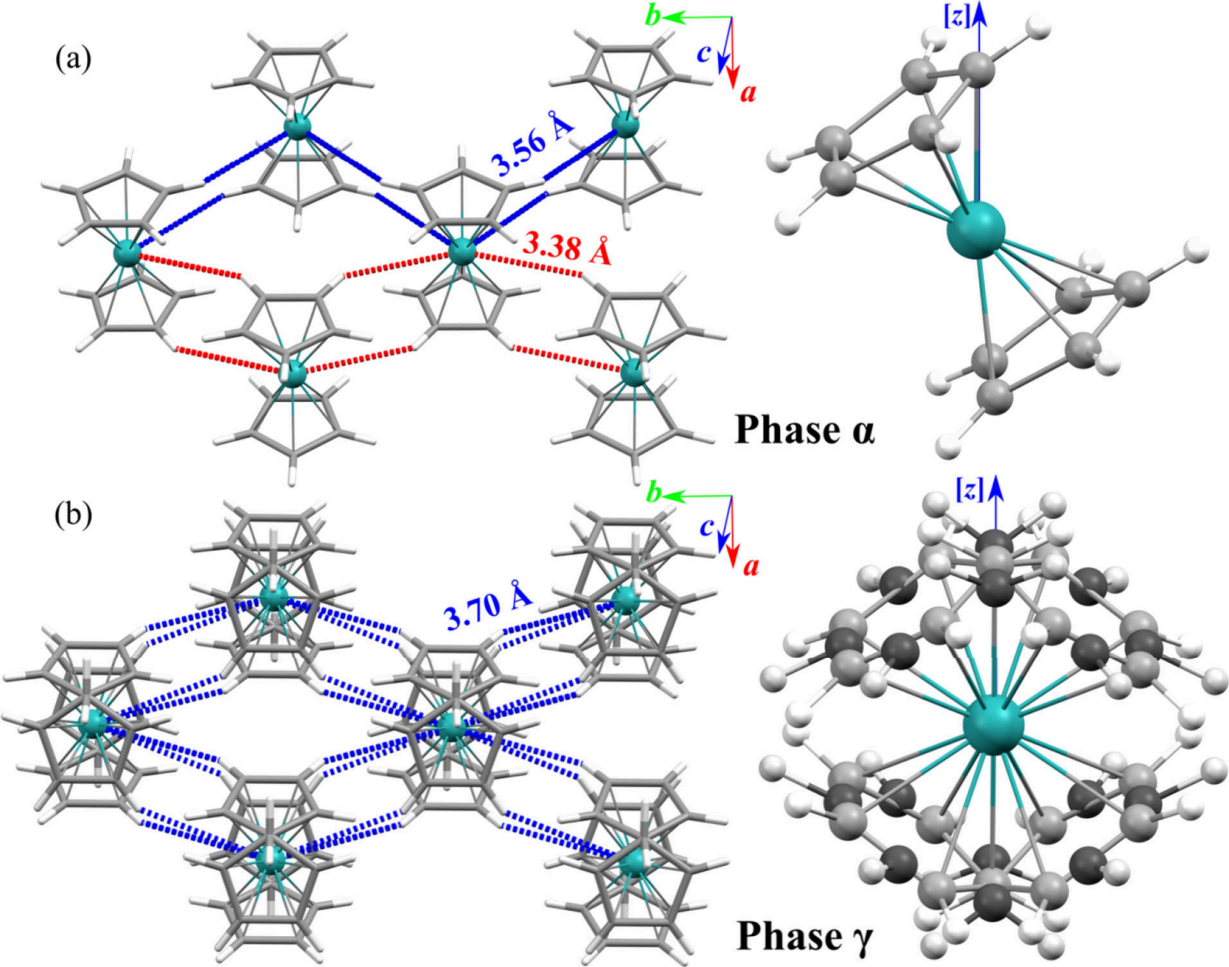
Autostereographic projections of ruthenocene phases (a) α and (b) γ and the ordered and disordered molecules inclined to the [*z*] direction by angle α_[*z*]_. The shortest CH···Ru distances are indicated by red and blue dotted lines to distinguish symmetry-independent contacts in phase α at 290 K, while all indicated contacts in phase γ at 400 K are equivalent. One set of sites has been skipped in phase γ (b) for clarity. The components of disorder are deconvoluted in Figure S7.

The radial distribution of the electron density of carbon atoms in the Cp ring as a function of temperature through the ordered and disordered phases α and γ of ruthenocene is plotted in Figure 5. It shows that some superposition of the distributions is present in phase α, even at 100 K, but it becomes significant at 200 K. This superposition of the densities of incoherently vibrating neighboring atoms, clearly manifested at still higher temperatures, is consistent with the very wide pretransitional entropy increase observed in the calorimetric measurements (stretching for about 150 K), as well as with the low *E*_p_ barrier for the Cp ring rotations. This electron-density distribution in phase α, where the positions of atoms (ordering of the Cp rings) is beyond doubt, illustrates the coexistence of two aspects of the atomic positions: those long-range vibrations correlated consistently with the crystal symmetry and were thus traceable through the Fourier analysis, yielding fixed mean positions of atoms, and the uncorrelated thermal vibrations, strongly diminishing the form factors as a function of the scattering angle θ. Thus at 296 K, despite the well-defined mean atomic centers (at φ=*n*·72°, where *n* = 0, 1–5), the overlap region (at φ = *n*·72° + 36°) accounts for about half of the electron density (instantaneous atomic positions). At 350 K, on approaching the transition temperature, the overlap region accounts for about 75% of the atomic distribution around the ring, which testifies that the large-amplitude torsional vibrations are present, while each C-atom is clearly located in a single position, and no other sites are present.

**Figure 5:**
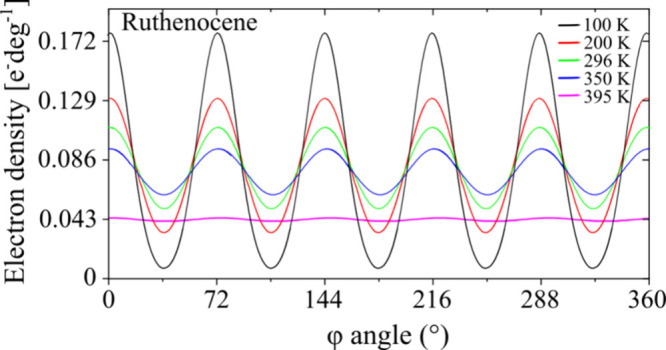
Electron density of carbon atoms radially distributed along the cyclopentadienyl ring as a function of temperature in ruthenocene phases α (100–350 K) and γ (395 K). These distributions were obtained by adding the probability distribution functions of the C atoms vibrating harmonically about their equilibrium positions, with the amplitudes derived from the anisotropic atomic displacement parameters in the directions tangential to the Cp ring. The amplitudes of the tangential components of mean atomic displacements were averaged for all atoms in the Cp ring and converted to the radial distribution. For phase γ, the atomic displacements were averaged separately for the disordered sites A and B; the common mean of the tangential components for atoms in A and B sites yielded the perfectly flat distribution (cf. Figure S9).

Above the transition to phase γ, apart from the seesaw disorder, the practically continuous distribution of electrons of carbon atoms, in light of the 4-fold increased number of states in phase γ compared to phase α, can be interpreted as the strong thermal uncorrelated vibrations of atoms (rings) around their disordered mean (long-range correlated) positions at sites A (φ = *n*·72°) and B (φ = *n*·72° + 36°). This model is consistent with the Fourier map analysis, which reveals the positions of the electron densities of the disordered rings at two mean positions A and B radially separated by 36° along the ring circumference and their successful anisotropic refinement. This Fourier analysis confirms the long-range correlation of the atomic positions in the rings in the crystal (cf. Figure S8). It should be stressed that the continuously nearly constant distribution of the electron density resulting from the summation of atoms in both sites A and B and their thermal vibrations is the projection of all the real crystal structure onto the independent part of the unit cell. The alternative interpretation of the continuous distribution of electrons in the Cp ring is its free rotation, but it would imply a much higher entropy change,^38^ and no long-range correlations of atomic positions in the crystal would yield not the mean atomic sites in the Fourier maps but their continuous circular distribution. Therefore it is plausible that the ring positions persist to be correlated in phase γ, most likely through the hindered rotations of the ring additionally complicated by the seesaw jumps, and the strong thermal motions of the atoms lead to the atomic-density distribution overlaps. It should be stressed that in the structural refinements of all the structures in this work we have applied the harmonic model of atomic vibrations. It is adequate for the low-amplitude vibrations, but for the strongly vibrating Cp rings at high temperatures, such as those in phases α and γ of ruthenocene and osmocene, considerable anharmonic contributions are expected. The measurements of the anharmonic vibrations are hampered due to the limited resolution at high temperatures and the presence of strong X-ray scatterers in these structures. The theoretical computations based on the harmonic approximation for isolated molecular dimers predict hindered-to-free rotor transitions at 220 K in the ferrocene dimer, at 270 K in the ruthenocene dimer, and at 350 K in the osmocene dimer.^26^

It should be also noted that the vibrations and disorder of Cp rings was thoroughly investigated for ferrocene, nickelocene, and other metallocenes.^11,25,26,39–45^ The primary concern was the distinction between the Cp ring disorder as the result of the dynamic motion of either rigid *M*Cp_2_ molecules or their internal torsions. The vibrational spectroscopy, inelastic neutron scattering, and theoretical computations indicate the prevailing contribution of the intramolecular torsions. Most of these experiments were performed for the low and ambient temperatures only, except for one experiment on nickelocene at 350 K.^11^

The seesaw reorientations of the whole molecule about axis [*y*] are by angle α_[z]_ = 30.32(2)° for ruthenocene at 400 K and 30.42(2)° for osmocene at 427 K. It is remarkable that angle α_[z]_ between the molecular pseuo-*C*_5_-axis and the crystal axis [*z*] change only by less than 1° through all temperature range between 100 and 440 K for RuCp_2_ and OsCp_2_ in their ordered α phases and disordered γ phases (cf. Figure S6). This angle α_[*z*]_ approximates the angle between the *M*–C bond and the pseudo-*C*_5_ axis, which is consistent with the coupling of the seesaw tumbling and the Cp rings rotations. In fact, the seesaw movement of ring 1 from its site Cp1_A_ brings it to site Cp1_B_ (Figures 4 and S7), and a ring rotation of 36° is needed to bring the ring into the new Cp1_A_ position. Likewise, the seesaw movement connects sites C2_A_ with C2_B_ and C2_B_ with C2_A_.

We have shown recently that the new high-pressure phase β of ruthenocene and the new phase β of osmocene were stabilized by anagostic CH···*M* bonds.^23,24^ The theoretical computations of the charge distribution on the surface of FeCp_2_, RuCp_2_ and OsCp_2_ molecules^24^ show that the magnitude of electronegative potential around the metal site grows in the sequence FeCp_2_ < RuCp_2_ < OsCp_2_, which make the H-acceptor capabilities grow in the same sequence: the lowest for FeCp_2_ and the largest for OsCp_2_. Most importantly, the computations showed that the magnitudes of the negative electrostatic potential on the molecular surface about the metal atoms are significantly larger for the eclipsed conformers and that the formation of anagostic bonds imposes steric hindrance on the staggered conformers. The electrostatic-potential magnitudes on the molecular surface about the *M* cation and the intermolecular H···H distances involving the closest neighbors of the H-atom involved in the CH···*M* bond are plotted in Figure 6. Thus, the close intermolecular H···H contacts favor the eclipsed conformers, too. The weak H-acceptor capability of the Fe-site in ferrocene and the absence of short CH···Fe contacts coincides with the domination of the *p*-*T* diagram of ferrocene by the disordered and staggered phases I, I′, and I″. Due to the temperature-agitated strong vibrations and rotations of Cp rings between the eclipsed and staggered conformations, the α phases of ruthenocene and osmocene are destabilized, and they both transform to the γ phases.

**Figure 6:**
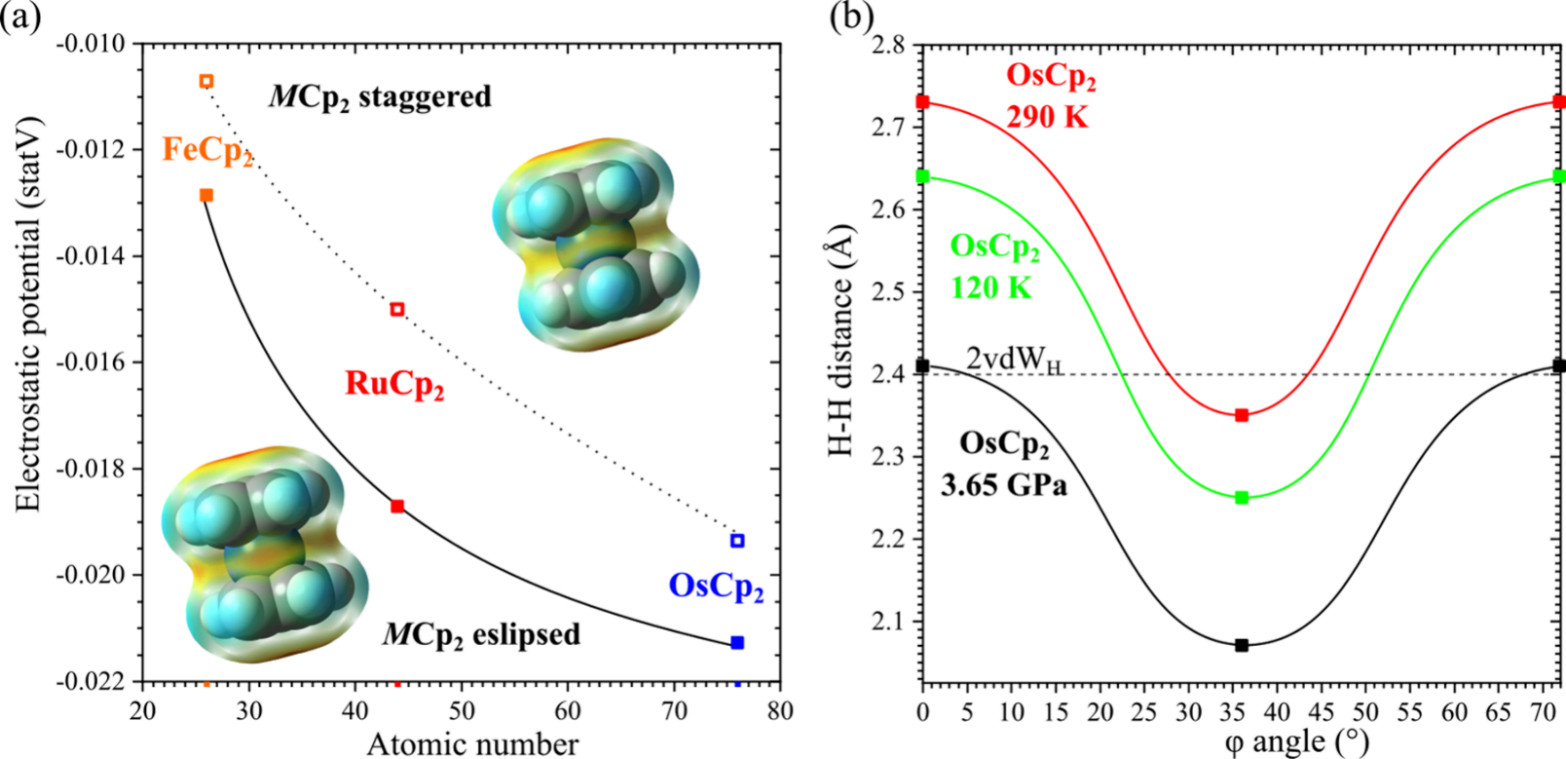
(a) Electrostatic potential on the molecular surface about the metal in ferrocene, ruthenocene and osmocene molecules; the insets show the electrostatic potential calculated for the molecule of osmocene in the staggered (top) and eclipsed (bottom) conformations. (b) Intermolecular H···H distances of the H atom involved in the anagostic bond to the closest H atom of the Cp ring in the experimental osmocene structures (eclipsed) and calculated for the other conformers.

The dynamic Cp-ring disorder (independent pseudo-*C*_5_ rotations in the disordering mode (ii) in γ-RuCp_2_ and γ-OsCp_2_ phases) results in equal populations of the staggered and eclipsed conformers. However, the staggered conformation hinders the access to the *M* cation, and the anagostic interactions present in phases α and β are broken. The transitions of α-ruthenocene and α-osmocene to the γ phases increases the lengths of anagostic CH···*M* contacts by over 0.2 Å (*cf*. Figure S10), i.e., significantly beyond the sums of the van der Waals radii of H and Ru (3.33 Å) and H and Os (3.36 Å).^46,47^ The direction of these contacts is close to the [*y*] direction, which is the axis of the seesaw motion (i), and therefore the increased CH···*M* distance corresponds to the largest elongation of the crystal along [*y*] on approaching the γ phase. The energy of breaking the CH···*M* bonds, the connected crystal expansion, and the entropy associated with the disorder all contribute to the considerable energy required for transforming the structures of ruthenocene and osmocene to phases γ. The plot in Figure 7 illustrates this relationship between the transition temperatures to the disordered phases with the CH···*M* distances, showing their essential role in controlling the molecular conformation.

**Figure 7:**
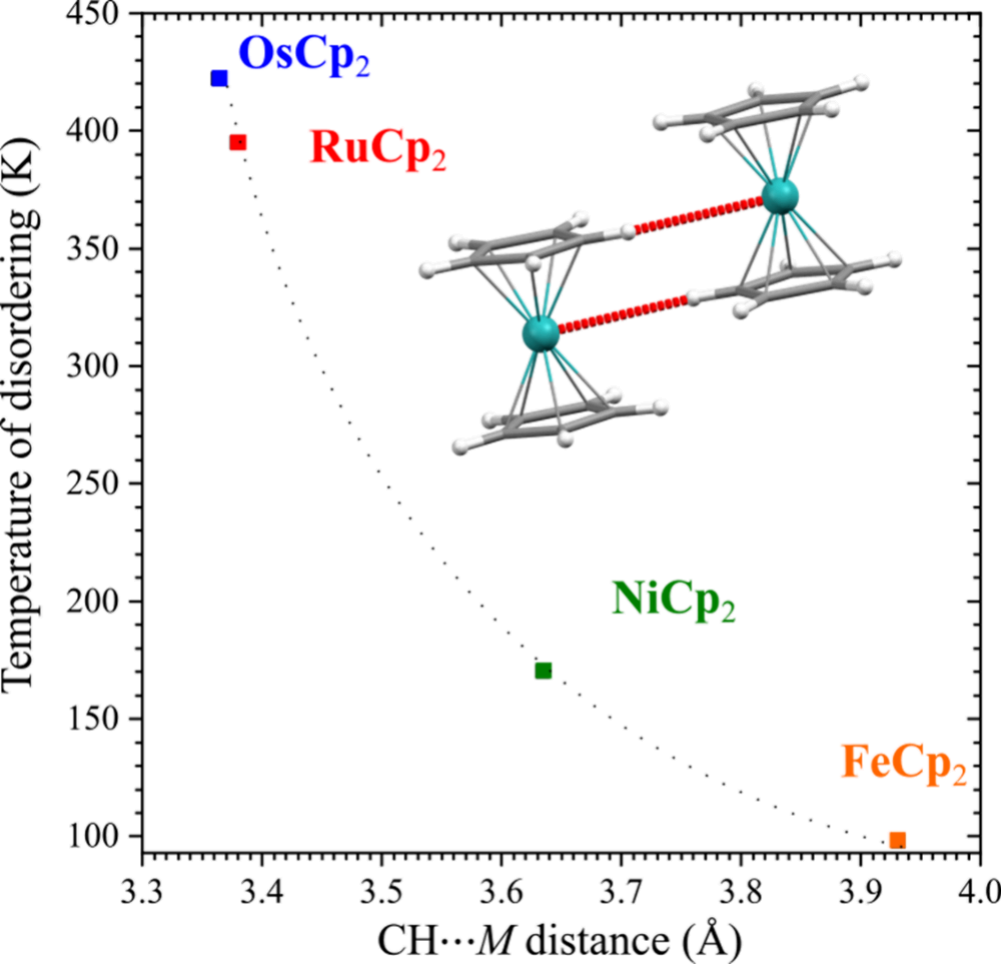
Temperature triggering conformational disordering in prototypic metallocenes as a function of the shortest CH···*M* distance, measured under ambient conditions (293 K/0.1 MPa).

We conclude that the new disordered phases of ruthenocene and osmocene revealed in this study widen a common landscape of transformations of prototypic metallocenes. Their unique sandwiched structure and axial *M*-Cp bonds result in conformational and dynamic properties inspiring the construction of molecular machines,^48,49^ not to mention other multiple applications of metalocenes. According to our study, the rotations of Cp rings and their seesaw movements can be thermally activated in ruthenocene and osmocene. To our knowledge, the seesaw disordering has not been considered so far for metallocenes, while there are intense studies of the nature of the Cp-ring rotations around the molecular *C*_5_ axis. We established that in the solid state, the anagostic bonds CH···*M* are responsible for the strong stabilization of the eclipsed conformers in ruthenocene and osmocene phases α and β. This conclusion is consistent with the H-acceptor capability increasing in the series FeCp_2_ < RuCp_2_ < OsCp_2_,^24^ which is also illustrated by the series of CH···*M* distances observed in the crystals of these compounds and the reverse correlation of CH···*M* distances with the temperatures required for triggering the conformational disorder. Other factors, like the molecular mass and Cp1–Cp2 ring distance, can be also significant, as suggested by the absence of the eclipsed phase in NiCp_2_ and the ordering of its Cp rings in the staggered conformation in phase I′, which for nickelocene extends to ambient-pressure low-temperature regions where ferrocene enters the eclipsed phase III. However, the role of anagostic bonds for the eclipsed conformers in ruthenocene and osmocene has been confirmed by their new γ phases.

## Experimental Methods

Differential scanning calorimetry (DSC) measurements were performed with a Q20000 calorimeter (TA Instruments), and DSC cooling and heating runs were measured for polycrystalline OsCp_2_ and RuCp_2_ samples at a temperature change rate of 10 K/min. The indium standard was used for the temperature and enthalpy calibration, whereas synthetic sapphire was used for the specific heat calibration.

Single-crystal (SCXRD) and powder (PXRD) X-ray diffraction measurements were conducted for the samples sealed in thin-wall glass capillaries to prevent sublimation (cf. Supporting Information). Agilent Xcalibur Atlas and Gemini A Ultradiffractometers, graphite-monochromated Mo Kα radiation, and an Oxford Cryostream Plus attachment were used to collect the low- and high-temperature X-ray diffraction data for all measurements for osmocene and for γ-ruthenocene at 400 K, while low- and high-temperature X-ray diffraction data for ruthenocene where collected with graphite-monochromated Cu Kα radiation on a Bruker D8 Quest diffractometer, also equipped with an Oxford Cryostream Plus attachment. Detailed experimental and crystal data are presented in Tables S1–S3.

The observations of the sample crystals undergoing the phase transitions for ruthenocene at 394.0 K and osmocene at 421.5 K were conducted with an Olympus MVX 10 microscope equipped with a digital CCD camera.

The SCXRD experiments and preliminary data reduction were performed with the CrysAlis software from Rigaku Oxford Diffraction and Apex III from Bruker.^50,51^ The SCXRD yielded the structures of RuCp_2_ and OsCp_2_ phases γ, solved by direct methods using Shelxt^52^ and refined by full-matrix least-squares on *F* ^2^ values with Shelxl,^52^ operated through Olex 2.^53^ The H-atoms were located at idealized positions (C–H distance 1.0 Å) and their isotropic displacement parameter *U*_iso_ = 1.2 *U*_eq_ of their carriers. The high-temperature structures of ruthenocene and osmocene above *T*_c_ are disordered in a complex way involving two modes of seesaw tumbling and Cp ring reorientations by 36°, as described in the text and Supporting Information (cf. Figures 3, 4, S7, and S11); details of the refinements of the disordered structural models are given in the Supporting Information. The structures of ruthenocene and osmocene (2420348–2420357 and 2444809–2444813) have been deposited with the Cambridge Crystallographic Database Centre. The copies can be obtained free of charge on request from www.ccdc.cam.ac.uk.

The unit-cell parameters at 370 and 380 K for ruthenocene, as well as those 370, 400, 410, and 420 K for osmocene, were obtained by fitting the reflections in the PXRD patterns (marked with squares in the Figure 2) with program FullProf^54^ implemented in the Match! software.^55^

## Supplementary Material





## Notes

Differential scanning calorimetry (DSC) heat flow plot for ruthenocene and osmocene, averaged carbon vibrational ellipsoid parameter U_1_ tangential to the Cp rings for ruthenocene and osmocene, crystal structures of α- and γ-RuCp_2_, deconvolution of a disordered molecule, angle α_[*z*]_ as a function of temperature for RuCp_2_ and OsCp_2_, CH···*M* distance for ruthenocene and osmocene as a function of temperature, discussion of unit-cell parameter changes, and entropy change calculation (PDF)
Transparent Peer Review report available (PDF)
