# Supplementary material for: New Osmocene and Ruthenocene Phases Reveal the Common Conformational Behavior Regulated by Anagostic Bonds in Prototypical Metallocenes
Source: J Phys Chem Lett. 2025 Jun 3;16(23):5755–62. doi: 10.1021/acs.jpclett.5c00686 (PMC12169653; doi:10.1021/acs.jpclett.5c00686)
Supplement: Supplementary file 1 [file jz5c00686_si_001.pdf]

New Osmocene and Ruthenocene Phases Reveal the Common Conformational Behavior Regulated by Anagostic Bonds in Prototypical Metallocenes

Ida Moszczyńska,[a] *Marek Szafrański,*[b] *Andrzej Katrusiak*[a*]

[a] Department of Materials Chemistry, Faculty of Chemistry, Adam Mickiewicz University, Uniwersytetu Poznańskiego 8, 61-614 Poznań, Poland

[b] Faculty of Physics, Adam Mickiewicz University, Uniwersytetu Poznańskiego 2, 61-614 Poznań, Poland

Supporting Information

Experimental

Entropy change calculation

The entropy change $\Delta \boldsymbol{S}$ was calculated by the signal integration above the baseline, according to formula:

$$\Delta S = \int_A^B \frac{Cp(T) - Cp^0(T)}{T}\, dT,$$

where $Cp^0(T)$ is the baseline temperature evolution.

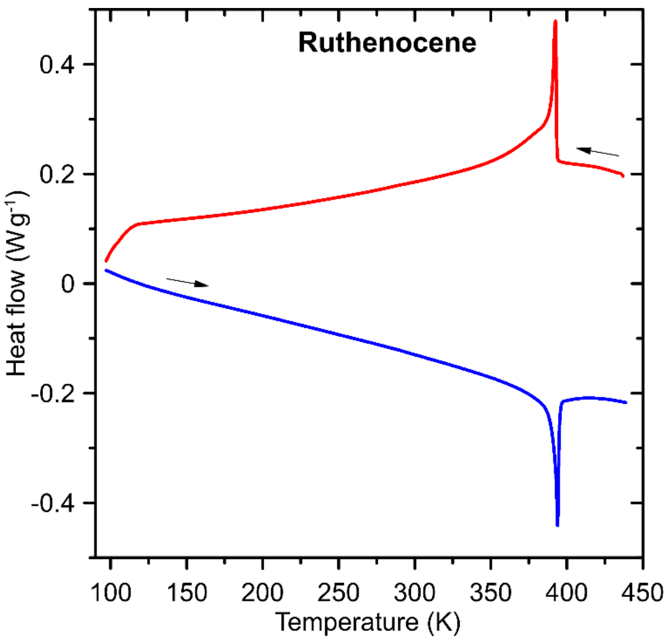

Figure S1. DSC heat flow plot for ruthenocene in the temperature range from 100 to 440 K. Heating and cooling runs are indicated plotted in blue and red, respectively.

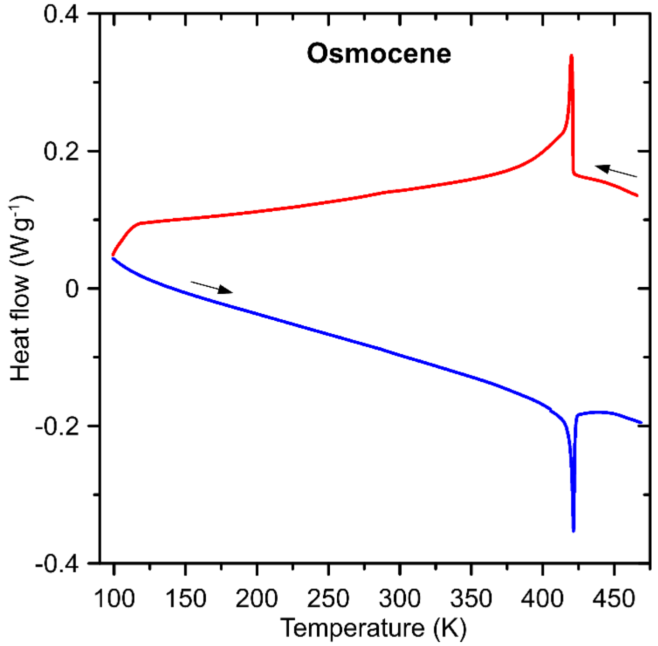

Figure S2. DSC heat flow plot for osmocene in the temperature range from 100 to 470 K. Heating and cooling runs are plotted in blue and red, respectively.

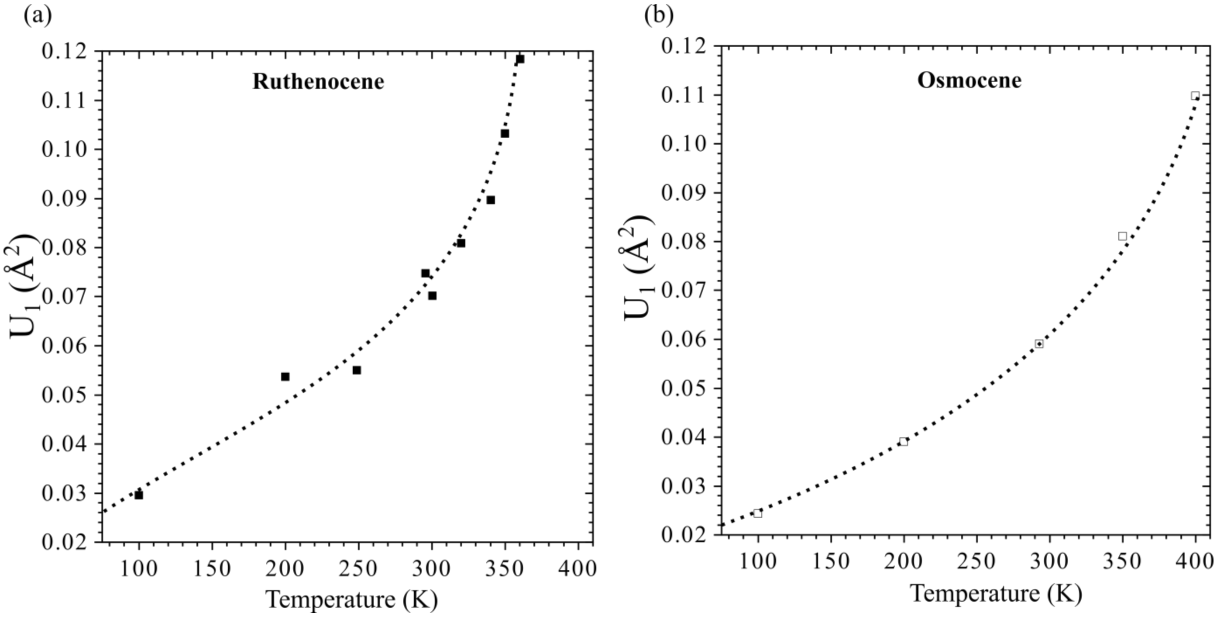

Figure S3. Mean square semiaxes U_1 of atomic displacement ellipsoids, averaged for all carbon atoms in the Cp rings.

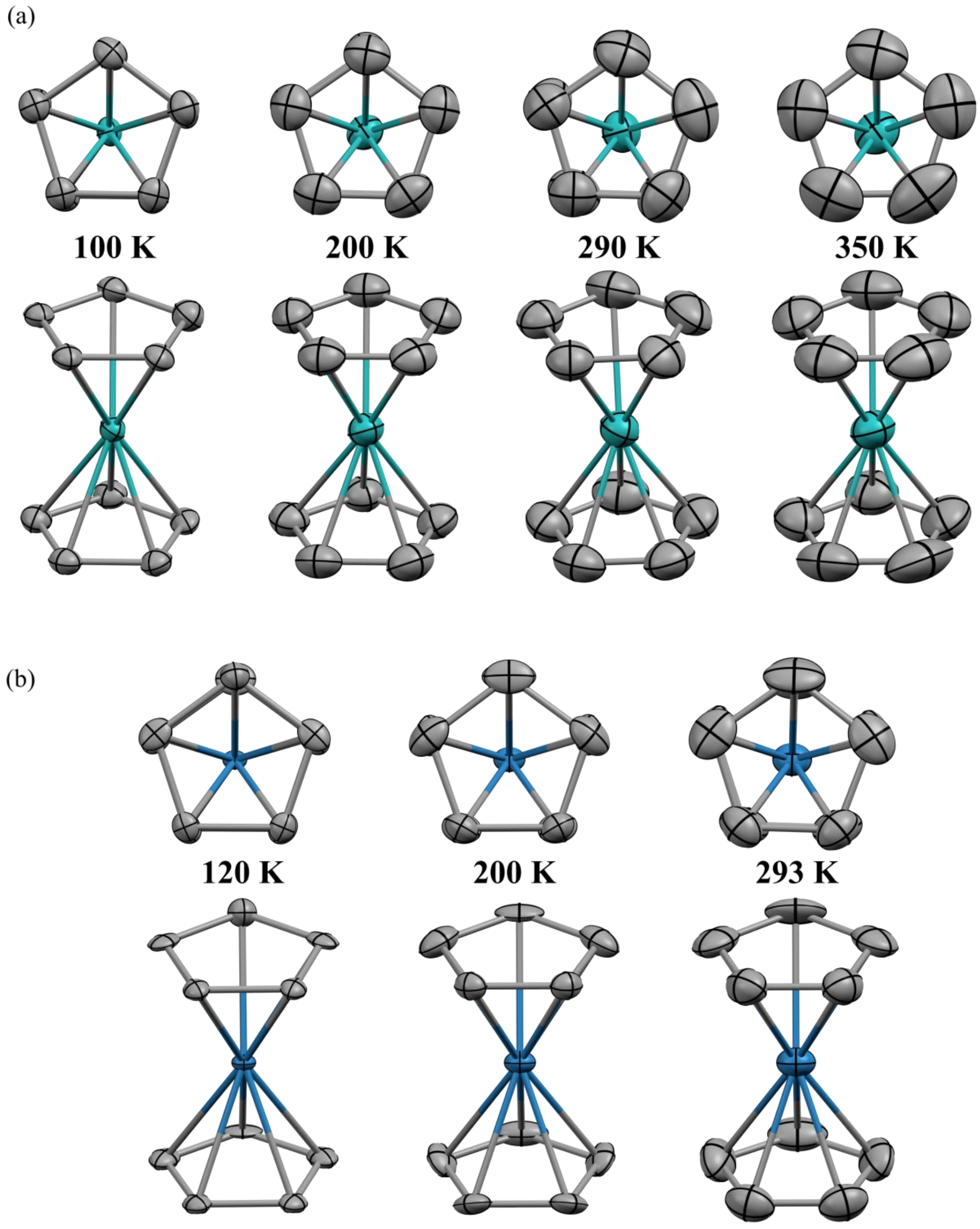

Figure S4. Atomic ellipsoids in (a) α-ruthenocene and (b) α-osmocene molecules; all drawn at the 50% probability level.

Single-crystal X-ray diffraction (SCXRD) measurements and structure refinements

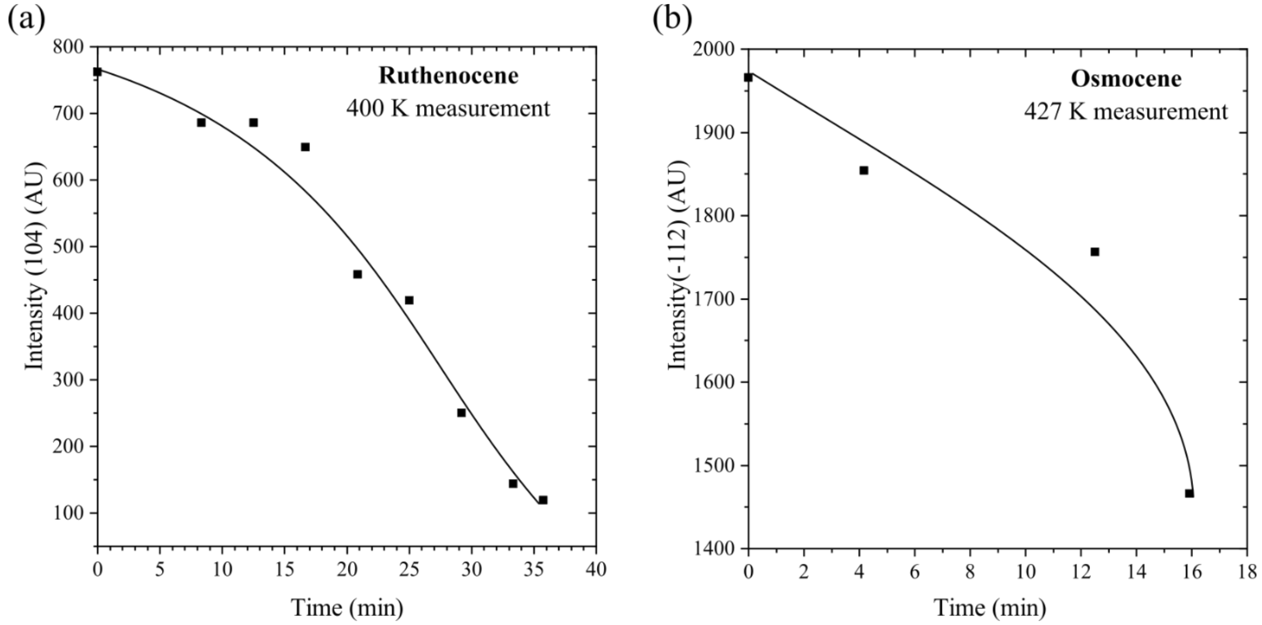

Figure S5. Intensity of control reflections as a function of time during the SCXRD data collections for (a) ruthenocene at 400 K; and (b) osmocene at 427 K.

Although the disordered C atoms were easily located in the Fourier maps, their free refinement led to distorted bond lengths and unrealistic differences in ADPs. Hence a number of restrains has been applied. The different number of restrains in the γ phase is a consequence of different quality of the measured datasets. The structural models of $RuCp_2$ at 393 K and 400 K could be consistently refined by imposing 95 restrains. The number of restrains involving only the C atoms was 80, and included the lengths of 3 independent C-C bonds in one site and 3 in the other (AFIX 56) and (SADI) and atomic displacement parameters (ADPs) for the 3 independent C atoms in each of their two disordered sites (SIMU); for the C atoms these restrains were also applied for the model of the γ-$OsCp_2$ at 427 K structure, additionally the ADP of one C atom was restrained with ISOR 0.01. The positions of H-atoms in the disordered rings required additional restraints to be applied in the structural models. Essentially, the H-atoms were located to fulfil the idealized positions in the Cp plane and with the C-H bond length close to 1.0 Å. Command SADI was used to average the length of the C-H bonds.

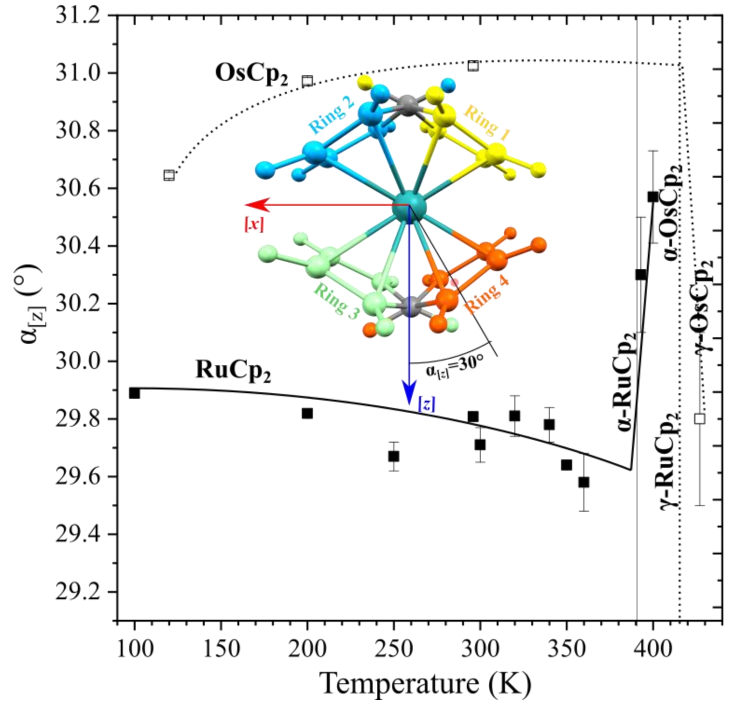

Figure S6. Molecular tilt in $RuCp_2$ (full symbols) and $OsCp_2$ (open symbols) between the pseudo C_5 axis of the molecule and [*z*] axis (angle $\alpha_{[z]}$) as a function of temperature, through phases α and γ. For the γ phases the molecules become disordered at the ± $\alpha_{[z]}$ tilts, but only the positive values are plotted. In the inset, only the seesaw disorder and the Cp rings sites A are shown, while the Cp sites B are omitted for clarity (see the text, cf. Figure S7).

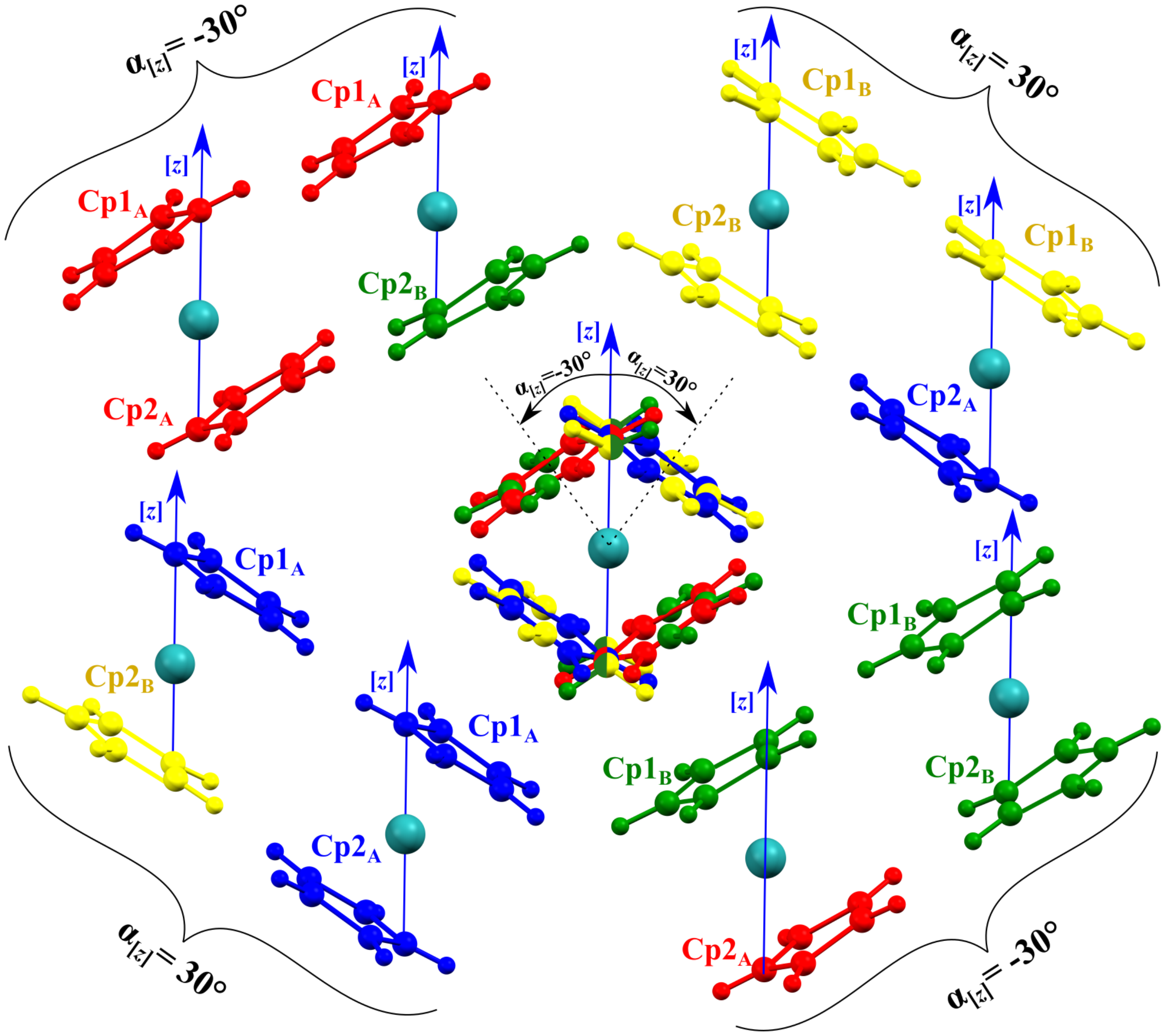

Figure S7. Deconvolution of disordered rings in molecule of ruthenocene in phase γ. The color code distinguishes the different sites of the disordered molecule.

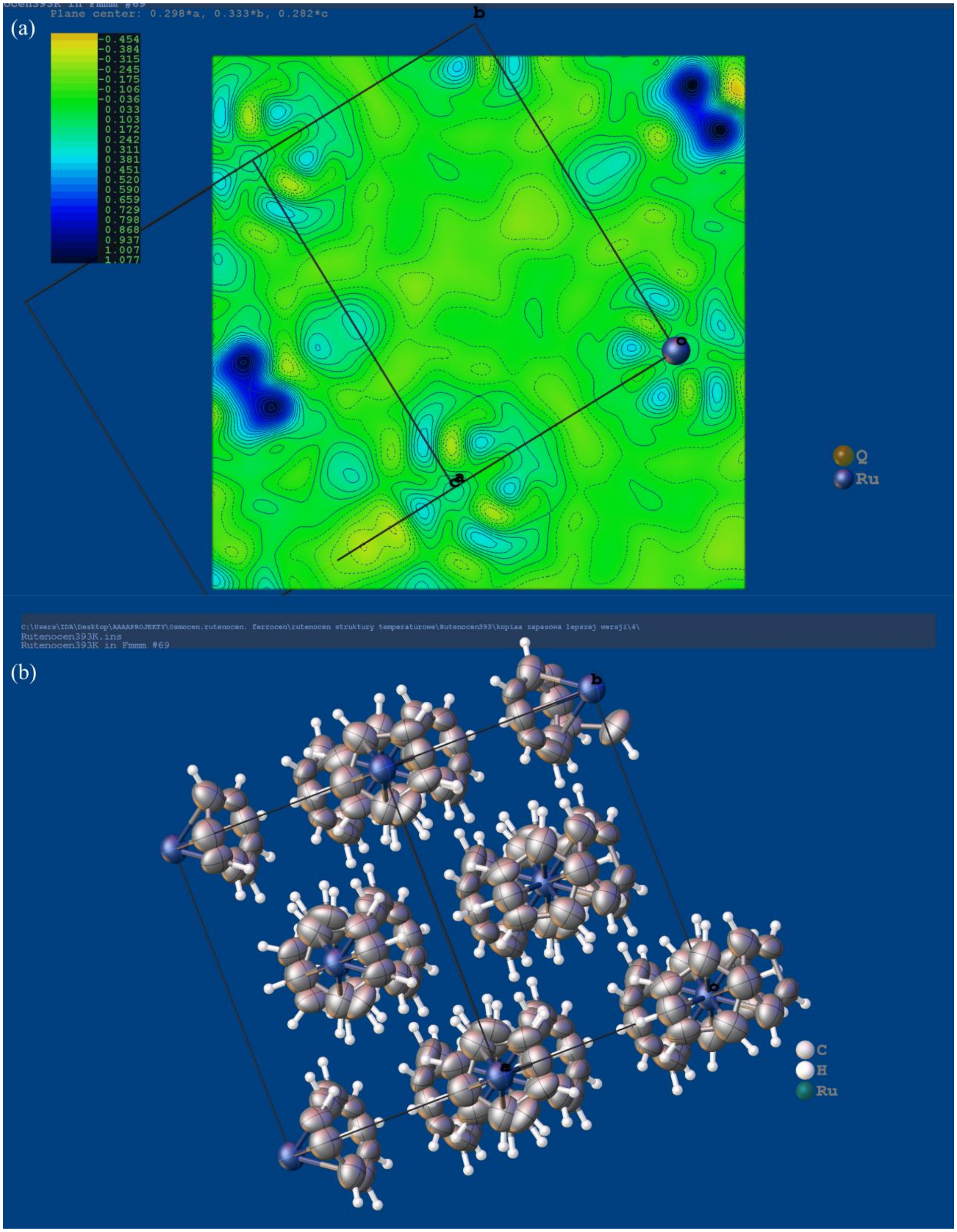

Figure S8. A section through the difference Fourier map calculated for ruthenocene phase γ at 393 K, positioned in the orientation to visualize the distribution of electron density along the Cp ring (a); to facilitate the inspection of this Fourier map, the crystal structure in the same orientation is shown (b).

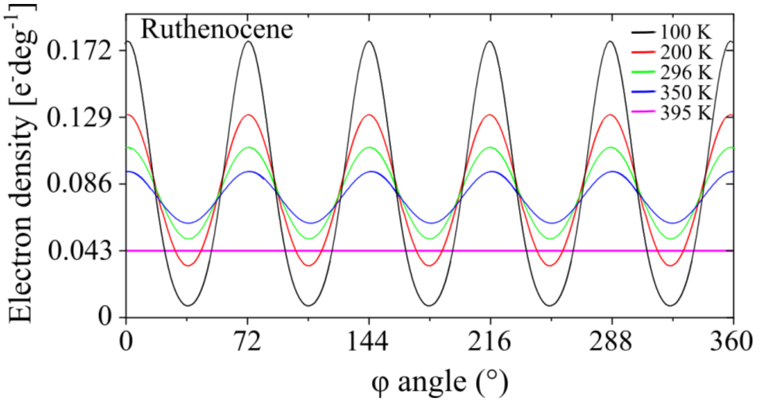

Figure S9. The electron density of carbon atoms radially distributed along the cyclopentadienyl ring as a function of temperature in ruthenocene phases α (from 100 to 350 K) and γ (at 395 K). This figure is nearly identical with Figure 5 in the main text, except for the density of phase γ at 395 K, where the atomic displacements were ***averaged together*** for the disordered sites A and B, yielding the perfectly flat distribution

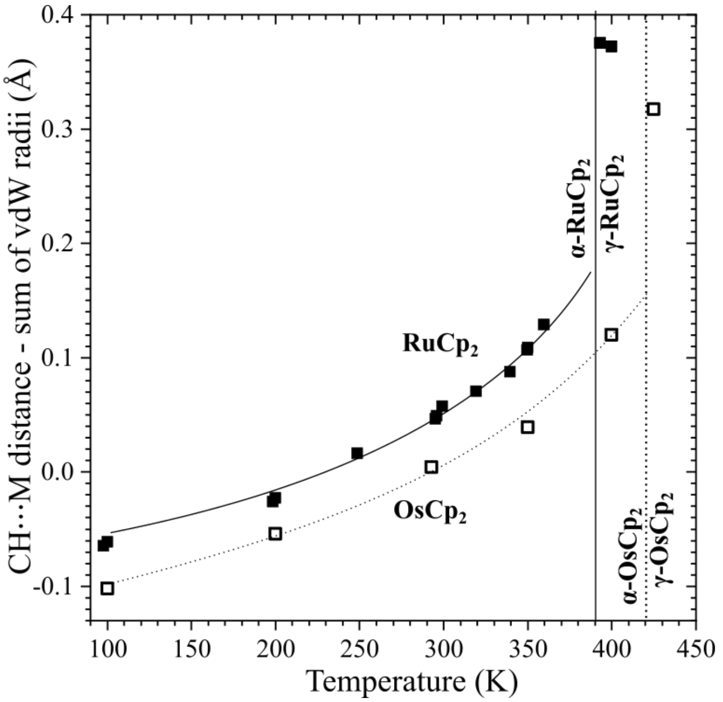

Figure S10. CH···*M* distance reduced by the sum of van der Waals radii[1,2] (r_{Os}=2.16 Å, r_{Ru}=2.13 Å, r_H=1.2 Å) plotted as a function of temperature. These differences (CH···Ru and CH···Os) are plotted with full and empty symbols, respectively. Vertical lines indicate the high temperature phase transitions of ruthenocene (full line) and osmocene (dotted line).

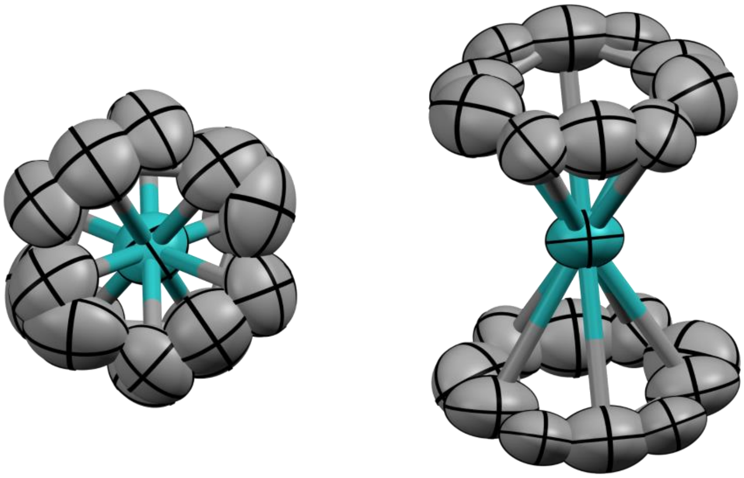

Figure S11. Two views of the disordered quarter-occupied rigs in γ-ruthenocene at 393 K, with the seesaw disorder and H-atoms eliminated for clarity. Atomic displacement parameters are drawn at the 50% probability level.

Table S1. Experimental and crystal data of ruthenocene and osmocene.

Compound	$RuC_{10}H_{10}$				$RuC_{10}H_{10}$	
Phase	α				α	
Temperature (K)	100(2)	200(2)	290(2)	350(2)	250(2)	300(2)
Formula weight	231.25				231.25	
Crystal color	Colorless				Colorless	
Crystal size (mm)	0.40×0.39×0.30				0.37×0.33×0.28	
Crystal system	Orthorhombic				Orthorhombic	
Space group	*Pnma*				*Pnma*	
Unit cell (Å) *a*	7.0038(2)	7.0518(2)	7.10970(10)	7.1599(2)	7.0804(2)	7.1085(3)
b	8.8208(3)	8.8865(3)	8.9752(2)	9.0648(2)	8.9355(4)	8.9803(4)
c	12.7630(5)	12.7761(4)	12.7934(3)	12.8054	12.7875(5)	12.7853(5)
Volume ($Å^3$)	788.49(5)	800.62(4)	816.36(3)	831.11(4)	809.03(5)	816.17(6)
Z/Z'	4/0.5	4/0.5	4/0.5	4/0.5	4/0.5	4/0.5
Density (g/cm^3)	1.948	1.918	1.882	1.848	1.899	1.882
Wavelength (Å)	CuK_{α}<br>1.54178	CuK_{α}<br>1.54178	CuK_{α}<br>1.54178	CuK_{α}<br>1.54178	MoK_{α}<br>0.71073	MoK_{α}<br>0.71073
Absorption (mm^{-1})	15.425	15.191	14.898	14.634	1.860	1.843
F(000)	456	456	456	456	456	456
2θ max (deg.)	144.534	144.086	144.656	144.732	57.45	57.356
Index ranges h_{min}/h_{max}	-8/8	-8/8	-8/8	-8/8	-8/8	-8/8
k_{min}/k_{max}	-10/10	-10/10	-10/10	-10/10	-11/7	-11/7
l_{min}/l_{max}	-13/15	-15/15	-15/15	-15/15	-12/16	-16/12
Refl. Collected	6236	3984	4136	4400	2722	2740
Refl. observed ($I>4\sigma_I$)	808	815	841	856	980	997
R(int)	0.0412	0.0452	0.0431	0.0413	0.0275	0.0303
Data/restraints/parameters	808/0/55	815/0/55	841/0/55	856/0/55	980/0/55	997/0/55
Goodness-of-fit on F^2	1.285	1.179	1.177	1.148	1.071	1.029
Final R_1 ($I>2\sigma_I$)	0.0470/<br>0.1368	0.0476/<br>0.1249	0.0409/<br>0.1038	0.0398/<br>0.0977	0.0330/<br>0.0725	0.0345/<br>0.0745
R_1/wR_2 (all data)	0.0472/<br>0.1370	0.0485/<br>0.1257	0.0434/<br>0.1052	0.0427/<br>0.0991	0.0449/<br>0.0800	0.0490/<br>0.0840

Table S1. (Continuation) Experimental and crystal data of ruthenocene and osmocene.

Compound	$RuC_{10}H_{10}$			$OsC_{10}H_{10}$		
Phase	α			α		
Temperature (K)	320(2)	340(2)	360(2)	120(2)	200(2)	293(2)
Formula weight	231.25			320.38		
Crystal color	colorless			colorless		
Crystal size (mm)	0.37×0.33×0.28			0.36×0.28×0.22		
Crystal system	Orthorhombic			Orthorhombic		
Space group	*Pnma*			*Pnma*		
Unit cell (Å) *a*	7.1231(3)	7.1402(3)	7.1603(5)	6.9838(2)	7.0292(3)	7.0848(3)
b	9.0060(4)	9.0363(4)	9.0759(7)	8.7716(4)	8.8301(4)	8.9122(5)
c	12.7896(6)	12.7917(6)	12.7952(7)	12.7472(5)	12.7683(6)	12.7883(7)
Volume ($Å^3$)	820(46)	825.33(6)	831.51(10)	780.88(5)	792.51(6)	807.47(7)
Z/Z'	4/0.5	4/0.5	4/0.5	4/0.5	4/0.5	4/0.5
Density (g/cm^3)	1.872	1.861	1.847	2.725	2.685	2.635
Wavelength (Å)	MoK_α 0.71073	MoK_α 0.71073	MoK_α 0.71073	MoK_α 0.71073	MoK_α 0.71073	MoK_α 0.71073
Absorption (mm^{-1})	1.834	1.823	1.809	16.249	16.011	15.714
F(000)	456	456	456	584	584	584
2θ max (deg.)	57.286	57.212	57.118	54.826	54.872	55.088
Index ranges h_{min}/h_{max}	-8/8	-9/8	-8/9	-4/8	-8/8	-8/8
k_{min}/k_{max}	-7/11	-11/7	-11/7	-9/11	-9/11	-10/11
l_{min}/l_{max}	-16/12	-12/16	-16/11	-16/14	-15/7	-15/14
Refl. Collected	2764	2786	2281	4042	3408	3492
Refl. observed ($I>4\sigma_I$)	1006	1013	954	885	878	896
R(int)	0.0283	0.0301	0.0369	0.0505	0.0462	0.0496
Data/restraints/parameters	1006/0/55	1013/0/55	954/0/55	885/12/56	878/0/56	896/0/56
Goodness-of-fit on F^2	1.056	1.046	1.021	1.078	1.070	1.001
Final R_1 ($I>2\sigma_I$)	0.0357/ 0.0797	0.0363/ 0.0806	0.0432/ 0.0975	0.0286/ 0.0620	0.0324/ 0.0677	0.0322 0.0550
R_1/wR_2 (all data)	0.0528/ 0.0914	0.0568/ 0.0947	0.0709/ 0.1149	0.0472/ 0.0689	0.0524/ 0.0758	0.0610 0.0645

Table S1. (Continuation) Experimental and crystal data of ruthenocene and osmocene.

Compound	$RuC_{10}H_{10}$	$RuC_{10}H_{10}$	$OsC_{10}H_{10}$
Phase	γ	γ	γ
Temperature (K)	393(2)	400(2)	427(2)
Formula weight	231.25	231.25	320.38
Crystal color	colorless	colorless	colorless
Crystal size (mm)	0.40×0.39×0.30	0.35×0.32×0.28	0.39×0.35×0.32
Crystal system	Ortorhombic	Ortorhombic	Orthorhombic
Space group	*Fmmm*	*Fmmm*	*Fmmm*
Unit cell (Å) *a*<br>*b*<br>*c*	7.2224(3)<br>9.2221(4)<br>12.7955(5))	7.2161(5)<br>9.2195(6)<br>12.7724(6)	7.1956(4)<br>9.1560(5)<br>12.7736(7)
Volume (Å^3)	852.25(6)	849.73(9)	841.56(8)
Z/Z'	4/0.5	4/0.5	4/0.5
Density (g/cm^3)	1.802	1.808	2.529
Wavelength (Å)	CuK_α<br>1.54178	MoK_α<br>0.71073	MoK_α<br>0.71073
Absorption (mm^{-1})	14.271	1.771	15.077
F(000)	456	456	584
2θ max (deg.)	144.138	57.256	56.992
Index ranges h_{min}/h_{max}<br>k_{min}/k_{max}<br>l_{min}/l_{max}	-8/8<br>-11/11<br>-15/15	-8/9<br>-12/12<br>-15/17	-9/8<br>-11/11<br>-17/10
Refl. Collected	1188	1483	785
Refl. observed (I>4σ_I)	255	316	295
R(int)	0.0380	0.0410	0.0364
Data/restraints/parameters	255/120/47	316/120/47	295/126/48
Goodness-of-fit on F^2	1.319	1.110	1.143
Final R_1 (I>2σ_I)	0.0431/ 0.0914	0.0332/ 0.0727	0.0377/ 0.0910
R_1/wR_2 (all data)	0.0431/ 0.0914	0.0355/ 0.0741	0.0399/ 0.0984

Table S2. Unit-cell dimensions of ruthenocene phases α and γ as a function of temperature.

Temperature (K)	*a* (Å)	*b* (Å)	*c* (Å)	method
**Phase α**				
100(2)	7.0038(2)	8.8208(3)	12.7630(5)	SCXRD (CuK$_\alpha$)
200(2)	7.0518(2)	8.8865(3)	12.7761(4)	SCXRD (CuK$_\alpha$)
250(2)	7.0804(2)	8.9355(4)	12.7875(5)	SCXRD (MoK$_\alpha$)
290(2)	7.10970(10)	8.9752(2)	12.7934(3)	SCXRD (CuK$_\alpha$)
300(2)	7.1085(3)	8.9803(4)	12.7853(5)	SCXRD (MoK$_\alpha$)
320(2)	7.1231(3)	9.0060(4)	12.7896(6)	SCXRD (MoK$_\alpha$)
340(2)	7.1402(3)	9.0363(4)	12.7917(6)	SCXRD (MoK$_\alpha$)
350(2)	7.1599(2)	9.0648(2)	12.8054	SCXRD (CuK$_\alpha$)
360(2)	7.1603(5)	9.0759(7)	12.7952(7)	SCXRD (MoK$_\alpha$)
370(2)	7.172	9.1118	12.799	PXRD (MoK$_\alpha$)
380(2)	7.18466	9.1256	12.1822	PXRD (MoK$_\alpha$)
**Phase γ**				
395(2)	7.2224(3)	9.2221(4)	12.7955(5)	SCXRD (CuK$_\alpha$)
395(2)	7.7172	9.241	12.793	PXRD (MoK$_\alpha$)
400(2)	7.2161(5)	9.2195(6)	12.7724(6)	SCXRD (MoK$_\alpha$)
402(2)	7.2276	9.2271	12.778	PXRD (MoK$_\alpha$)
405(2)	7.198	9.24	12.8	PXRD (MoK$_\alpha$)

Table S3. Unit-cell dimensions of osmocene phases α and γ as a function of temperature

Temperature (K)	*a* (Å)	*b* (Å)	*c* (Å)	method
**Phase α**				
120(2)	6.9838(2)	8.7716(4)	12.7472(5)	SCXRD (MoK_α)
200(2)	7.0292(3)	8.8301(4)	12.7683(6)	SCXRD (MoK_α)
293(2)	7.0848(3)	8.9122(5)	12.7883(7)	SCXRD (MoK_α)
350(2)	7.1281(1)	8.9849(2)	12.8127(2)	SCXRD (CuK_α)
370(2)	7.1216	8.9851	12.8119	PXRD (MoK_α)
400(2)	7.1496	9.0406	12.8152	PXRD (MoK_α)
400(2)	7.1727(9)	9.0766(11)	12.8180(14)	SCXRD (CuK_α)
410(2)	7.16036	9.0606	12.8184	PXRD (MoK_α)
**Phase γ**				
420(2)	7.176	9.159	12.972	PXRD (MoK_α)
427(2)	7.1956(4)	9.1560(6)	12.7736(7)	SCXRD (MoK_α)

Changes of unit-cell parameters: discussion

The *c* parameters of the unit cells of ruthenocene and osmocene are least temperature-dependent in α phase, which is due to hydrogen bonds C-H···π stabilizing the α-$RuCp_2$ and α-$OsCp_2$ structures along the [001] direction (Figure 4b).[3,4] The shortening of parameter *c* above T_c of ruthenocene at 395 K and osmocene at 427 K can be connected with the increased vibrations of H-atoms and H-disordering in Cp rings.

In γ phases of $RuCp_2$ and $OsCp_2$ we only consider the conformations between parallel ring pairs, because angles (Cp1 centroid)-*M*-(Cp2 centroid) in metallocene molecules are highly unlikely to bend by 60°. Such an unlikely bending would be hindered by very close contacts between hydrogen atoms of rings Cp1 and Cp2, equal 1.2 Å for eclipsed and 1.7 Å for staggered conformation, i.e. distances drastically shorter than the sum of two van der Waals radii of H-atoms, 2.4Å.[2] Moreover, the energy of bent molecule is around 450 kJ/mol higher than the molecule of straight one.

(1) Bondi, A. Van Der Waals Volumes and Radii. *J. Phys. Chem.* **1964**, *68*, 441–451.

(2) Hu, S. Z.; Zhou, Z. H.; Robertson, B. E. Consistent Approaches to van Der Waals Radii for the Metallic Elements. *Z. Kristallogr.* **2009**, *224*, 375–383.

(3) Moszczyńska, I.; Katrusiak, A. Competition between Hydrogen and Anagostic Bonds in Ruthenocene Phases under High Pressure. *J. Phys. Chem. C* **2022**, *126*, 5028–5035.

(4) Moszczyńska, I.; Gulaczyk, I.; Katrusiak, A. Giant Deformation between Osmocene Phases Induced by Anagostic Bonds Promoted under High Pressure. *J. Phys. Chem. C* **2023**, *127*, 19250–19257.
