# Supplementary material for: New Osmocene and Ruthenocene Phases Reveal the Common Conformational Behavior Regulated by Anagostic Bonds in Prototypical Metallocenes
Source: J Phys Chem Lett. 2025 Jun 3;16(23):5755–62. doi: 10.1021/acs.jpclett.5c00686 (PMC12169653; doi:10.1021/acs.jpclett.5c00686)
Supplement: Supplementary file 2 [file jz5c00686_si_002.pdf]

jz-2025-00686a.R1

Name: Peer Review Information for "New Osmocene and Ruthenocene Phases Reveal the Common Conformational Behavior Regulated by Anagostic Bonds in Prototypical Metallocenes"

First Round of Reviewer Comments

Reviewer: 1

Comments to the Author

The authors compared the crystal-to-crystal thermal phase transitions of ruthenocene and osmocene and conducted a detailed disorder analysis of the gamma-phase of osmocene, which is reported in this paper. The paper is well-structured and clearly written, covering its composition, introduction, results, discussion, and references. It presents fundamental scientific insights into structural changes in molecular single crystals, making it an interesting read for researchers studying molecular crystals. Therefore, after addressing the following revisions, this paper can be recommended for publication in JPCL.

Comments:

1. In page 4, line 23. “It is even more puzzling that ferrocene has been so far the only known metallocene topochemically transforming between staggered and eclipsed phases.” Is this in the crystalline state? This statement requires references.

2. In Figure 2, the longitudinal and transverse axes should be labeled in English, as required by ACS journals. Additionally, the light green color is difficult to distinguish.

Reviewer: 2

Comments to the Author

Review of jz-2025-00686a

The (Cp)2M molecules are classics. Any new experimental result about their structures is an important contribution. The observed transition from the Pnma phase to a disordered Fmmm structure is surprising. The transition is well documented both crystallographically and thermally.

A picture showing the atomic ellipsoids (see illustrations below) would be a very useful addition to the supplementary material so that readers could assess the quality of the displacement ellipsoids, which look better than I might have expected. In making this figure I discovered that the Ru structures at 200 K and 350 K are rotated by 180° relative to those at 100 K and 290 K, which is unfortunate.

A detailed description of how the disorder in the Fmmm phases was sorted out is missing. It looks to me as if the electron density in the rings is probably more or less continuous (see attached illustration). Were other models tried but less successful? What assumptions were made? Large numbers of restraints were used (109, 64 and 85 for the three Fmmm structures). What were they, and why were they so diƯerent for the three structures?

I spent time looking at the model for the disorder in the Ru Fmmm structure as determined at 393 K. Even with all the restraints that were applied the variation of the C-C distances in the Cp ring is very large (0.13 Å for the ring of C1,C2,C3,C3,C2; 0.17 Å for the ring of C1',C2',C3',C2',C1'. I conclude that the quality of the model is only modest, although it might well be the best model possible.

I also looked carefully at the occupancy factors and concluded that the two essentially parallel orientations of the Cp rings have equal populations. The symmetry of the disordered molecule is mmm so that in each of the four Cp planes there are two orientations of equal occupancy. The authors have chosen to say that eclipsed and staggered molecules related by approximate inversion are disordered but I do not see how the possibility of two diƯerent orientations of eclipsed molecules can be ruled out. The diƯerence between those two possibilities (eclipsed and staggered conformations or two orientations of eclipsed molecules) is the correlation between the orientations of two

parallel rings on opposite sides of the metal. Bragg peaks provide no information about such correlation.

The H stoms seem to be in the plane of the C5 ring and to have C-H distances that are 1.00 Å at some temperatures and 0.98 Å at others (rather than being 0.9 Å as is stated in the text). In Fig. 3 the H...Ru distances are given to the nearest 0.001 Å, which seems excessive given the method used to position the H atoms.

Given the apparent impossibility of finding a model that is chemically satisfactory, the uncertainty about whether the molecular conformations are eclipsed or staggered, and the calculated H -atom positions I consider the detailed interpretation of the structures starting with Fig. 3 on pg. 9 unjustified. (The question of whether or not anagostic interactions are strong enough to account for a phase transition is not important to this conclusion.)

The experimental work reported in this manuscript is important and should be published after revision (see comments above and below), but the experimental work does not support some of the interpretation presented and some of the conclusions drawn (such as the importance of anagostic interactions).

Less important points:

In the TOC graphic the disorder is shown for only two of the four rings. The metal atom lies on the origin, where the site symmetry is mmm. All four rings are therefore disordered.

In Fig. S3 five points are shown for the Pnma Os structure but only three cifs are provided for that structure.

On pg 6 it says "Above the transition temperatures the *c* parameters display a strong negative thermal expansion, also observed for parameters *a* and *b* of ruthenocene and parameter *b* of osmocene." but I cannot find data for the change with T of the cell constants of the Fmmm structures.

In Table S1 should the “A” in the second row, second column be changed to an alpha?

In the cif the chemical name is wrong in the two blocks containing information about the Ru Fmmm structure.

Why were 36 restraints needed in the refinement of the Os Pnma structure at 120 K but not at 200 K?

Why do the numbers of variables for the three Fmmm structures (50, 47, 58) diƯer?

Ru, Pnma in order of increasing temperature

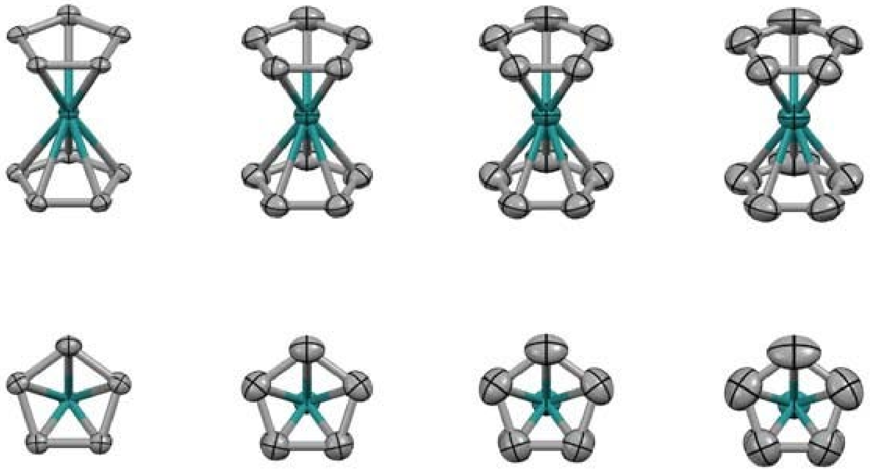

Os, Pnma in orderof increasing temperature

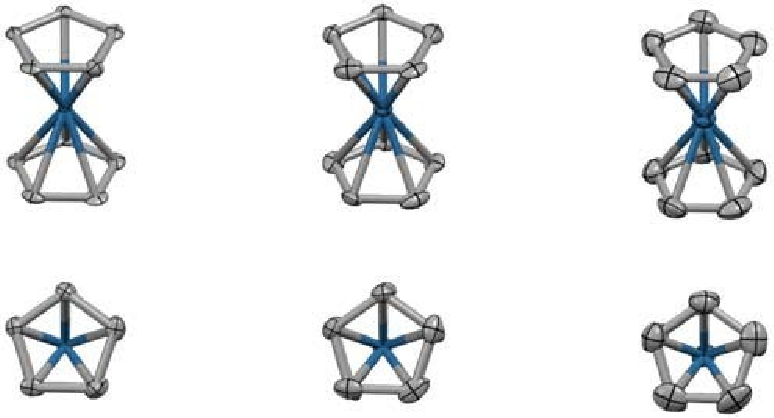

Ru, Fmmm at 393 K (atoms for one molecule only)

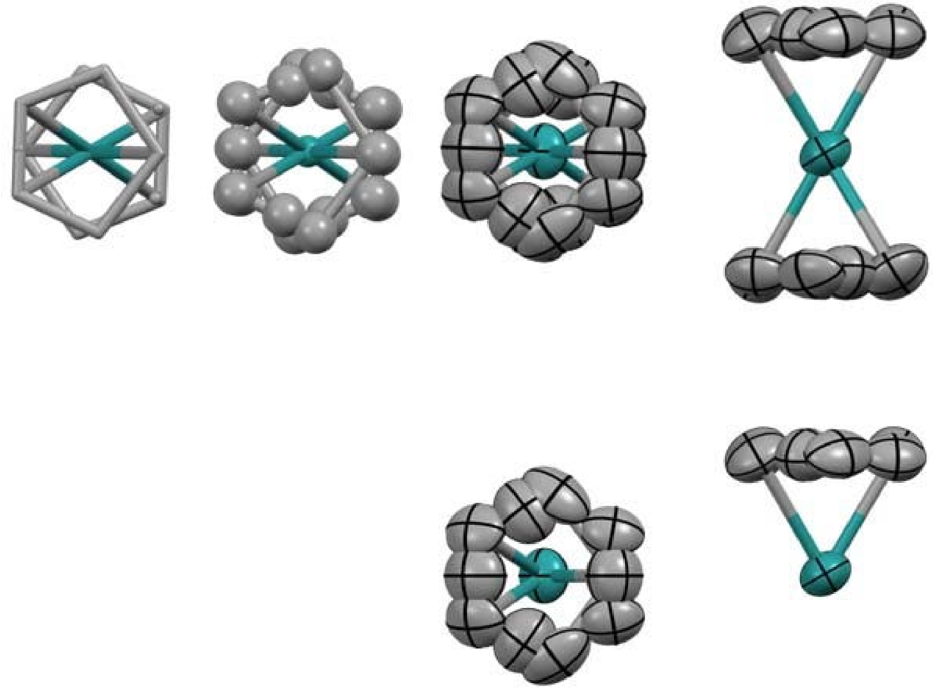

Author's Response to Peer Review Comments:

Dear Editor,

Thank you very much for your message of 28 March, 2025 and for the Referees' reports. We are very grateful to the referees for carefully reading our manuscript and for their comments. We have accordingly corrected the paper, which surely will increase its clarity and quality. We are submitting the revised versions of the manuscript and its Supporting Information. We have also revised the CIF files and additionally remeasured some of the high-temperature crystal structures, so additional new CIFs have been deposited in the Cambridge Crystallographic Database Centre.

Along with the manuscript and Si files, we are submitting their versions with all the changes highlighted, in order to facilitate reviewing the revisions – the submission is accompanied with the response to all referees' comments, enclosed here below.

With best regards,

Prof. Andrzej Katrusiak
Wydział Chemii UAM
Collegium Chemicum, Blok A, Biuro 1.19
Uniwersytet im. Adama Mickiewicza ul.
Uniwersytetu Poznańskiego 8 61-614 Poznań

URL hpc.amu.edu.pl
tel.: +48 618291590 fax:
+48 618291555
E-mail: katran@amu.edu.pl

Reviewer 1

The authors compared the crystal-to-crystal thermal phase transitions of ruthenocene and osmocene and conducted a detailed disorder analysis of the gamma-phase of osmocene, which is reported in this paper. The paper is well-structured and clearly written, covering its composition, introduction, results, discussion, and references. It presents fundamental scientific insights into structural changes in molecular single crystals, making it an

interesting read for researchers studying molecular crystals. Therefore, after addressing the following revisions, this paper can be recommended for publication in JPCL

1. In page 4, line 23. “It is even more puzzling that ferrocene has been so far the only known metallocene topochemically transforming between staggered and eclipsed phases.” Is this in the crystalline state? This statement requires references.

Authors respond: Thank You for your suggestion, we corrected the sentence into: “It is even more puzzling that ferrocene has been so far the only known metallocene crystal, which topochemically transforms between staggered and eclipsed phases.” We added the references revealing the transformation of ferrocene to the eclipsed state in phase III phase, a reinvestigation of this transformation, as well as a review about the conformational properties of metallocenes.

2. In Figure 2, the longitudinal and transverse axes should be labeled in English, as required by ACS journals. Additionally, the light green color is difficult to distinguish.

Authors respond: We apologize for this mistake! Thank you for noticing it – it has been corrected in the revised version.

Reviewer 2

The $(Cp)_2M$ molecules are classics. Any new experimental result about their structures is an important contribution. The observed transition from the Pnma phase to a disordered Fmmm structure is surprising. The transition is well documented both crystallographically and thermally.

1. A picture showing the atomic ellipsoids (see illustrations below) would be a very useful addition to the supplementary material so that readers could assess the quality of the displacement ellipsoids, which look better than I might have expected. In making this figure I discovered that the Ru structures at 200 K and 350 K are rotated by 180° relative to those at 100 K and 290 K, which is unfortunate.

Authors respond: We are grateful for this suggestion and we have added the drawings showing ADP ellipsoids in Figure S8. We have also corrected the orientation of molecules in the structures at 200 K and 350 K, so the asymmetric units are now consistent in all structural models of $RuCp_2$ and $OsCp_2$ attached as the Supporting Information and we have corrected the CCDC deposits, too.

2. A detailed description of how the disorder in the Fmmm phases was sorted out is missing. It looks to me as if the electron density in the rings is probably more or less continuous (see attached illustration). Were other models tried but less successful? What assumptions were made? Large numbers of restraints were used (109, 64 and 85 for the three Fmmm structures). What were they, and why were they so different for the three structures?

Authors respond: We fully agree with this comment, which concerns the apparent omission in our paper: the structural model should be clearly explained, which we have overlooked to do. We have added this information (see below) in the revised text.
Let us explain that the high-temperature experiments were challenging and we had to overcome several technical difficulties, like the sample sublimation and fixing its position. We failed in several tries, but finally we fixed the samples between cotton fibers gently pushed by a glass-rod nearly matching the inner diameter of the capillary; we additionally filled the space between the rod and capillary by Distal® (a two-component polymer glue). The capillaries were sealed by melting their ends in a micro torch: one capillary end was sealed first, then the cotton – sample – cotton – glass-rod were inserted and then the other end was melted together with the glass-rod end. In the temperature range close to the T_c and in the γ phase, these protections of the sample crystal were sufficient to collect one full data set (during 30 min) and to start the subsequent one – after its 5-to-10 minutes the sample crystal started to move due to its sublimation. During one 30-min data collection, the intensity of control reflections dropped by about 90%; the reflections intensities were accounted for this sublimation effect (see Figures below). Despite these experimental difficulties, the structures were solved straightforwardly by direct methods in Shelxs[1] and in the Fourier maps the electron density peaks clearly indicated the carbon atoms of the Cp ring disordered in four sites two due to the see-saw movement of molecules about the [y] axis and another two due to the Cp rings rotation by 36° about pseudo-C_5 molecular axis. This clear locations of 4-fold disordered Cp rings were consistent for the γ phases of ruthenocene and osmocene and their 4-fold disorder indicated by the entropy values close to Rln4 of the transitions between the α and γ phases (see the colorimetric results). All this information consistently confirmed the disordering mode, where the Cp ring jumps between two sites 36° apart about the pseudo-C_5 axis and between two sites one inclined by 60° to the other in the see-saw mode about the [z] axis.

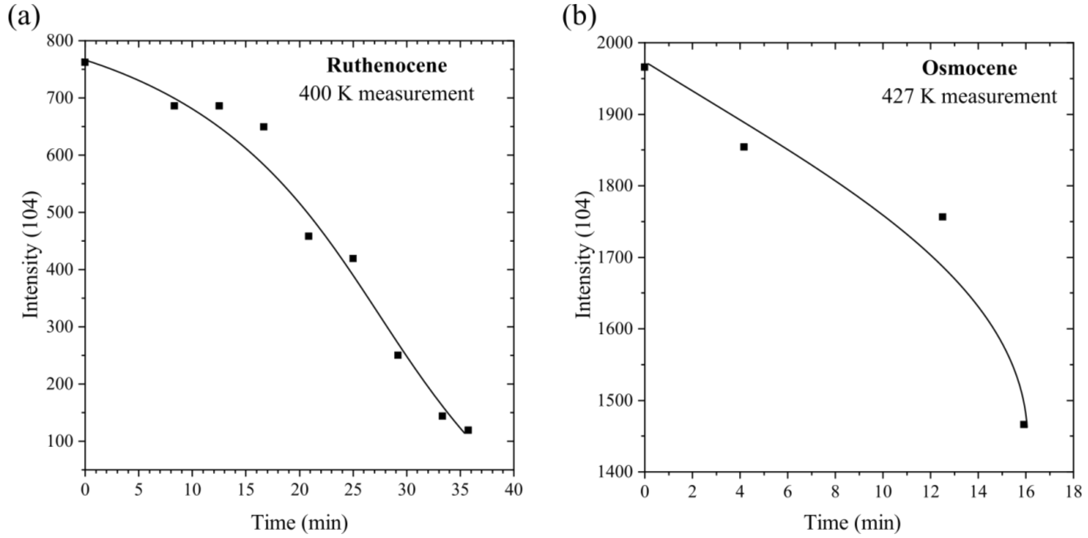

Although the disordered C atoms were easily located, their free refinement led to distorted bond lengths and unrealistic differences in ADPs. Hence a number of restrains has been applied. The different number of restrains in the γ phase is a consequence of different quality of the measured datasets. The structural models of $RuCp_2$ at 393 K and 400 K could be consistently refined by imposing 95 restrains. The number of restrain involving the C atoms only was 80, and included the lengths of 3 independent C-C bonds in one site and 3 in the other (AFIX 56) and (SADI) and atomic displacement parameters (ADPs) for the 3 independent C atoms in each of their two disordered sites (SIMU); for the C atoms these restrains were also applied for the model of the γ-$OsCp_2$ at 427 K structure, additionally the ADP of one C atom was restrained with ISOR 0.01. The positions of H-atoms in the disordered rings required additional restraints to be applied in the structural models. Essentially, the H-atoms were located to fulfil the idealized positions in the Cp plane and with the C-H bond length close to 1.0 Å. Command SADI was used to average the length of the C-H bonds.

It was our apparent omission that these experimental details were not explained in our manuscript – now we have added this information in the text and in the supporting Information.

3. I spent time looking at the model for the disorder in the Ru Fmmm structure as determined at 393 K. Even with all the restraints that were applied the variation of the C-C distances in the Cp ring is very large (0.13 Å for the ring of C1,C2,C3,C3,C2; 0.17 Å for the ring of C1',C2',C3',C2',C1'. I conclude that the quality of the model is only modest, although it might well be the best model possible.

Authors respond: We have imposed the SADI restraint on the C-C bonds, which reduced the variation of the C-C bond lengths to 0.005 Å for the ring of C1,C2,C3,C3,C2 and 0.011

Å for the ring C1’,C2’,C3’,C2’,C1’. This information has been added in the revised Supporting Information.

4. I also looked carefully at the occupancy factors and concluded that the two essentially parallel orientations of the Cp rings have equal populations. The symmetry of the disordered molecule is mmm so that in each of the four Cp planes there are two orientations of equal occupancy. The authors have chosen to say that eclipsed and staggered molecules related by approximate inversion are disordered but I do not see how the possibility of two different orientations of eclipsed molecules can be ruled out. The difference between those two possibilities (eclipsed and staggered conformations or two orientations of eclipsed molecules) is the correlation between the orientations of two parallel rings on opposite sides of the metal. Bragg peaks provide no information about such correlation.

Authors respond: From our experiment we can establish that the disordered sites are mmm (D_{2h}) symmetric and additionally to the seesaw disorder each half-occupied ring is further disordered in two 0.25-occupied positions one rotated to the other by 36°. If we eliminated the seesaw component (in a thought experiment), we would have half of the molecule disordered by 36° about its pseudo-5-fold axis. The symmetry of C_{2h} applies to both parts, rotated by 36° about the pseudo-C_5 axis one with respect to the other. This disorder is determined for one asymmetric ring part only (ring 1), and the positions of the other ring (ring 2) are obtained through the inversion axis or the 2-fold axis. Both these symmetry elements yield the staggered conformers. However, the inelastic neutron scattering, Raman spectra and the computational analysis show that the rotation of the “rigid” molecule by 36° are unlikely – practically impossible; these studies indicate that the rings rotate separately, and such a rotation of one ring has to involve the changes between the staggered and eclipsed conformers. We agree that this conclusion cannot be deduced from the X-ray diffraction experiment alone. We have improved this explanation in the text.

5. The H atoms seem to be in the plane of the C5 ring and to have C-H distances that are 1.00 Å at some temperatures and 0.98 Å at others (rather than being 0.9 Å as is stated in the text). In Fig. 3 the H...Ru distances are given to the nearest 0.001 Å, which seems excessive given the method used to position the H atoms.

Authors respond: Thank you for your suggestion, we have corrected the value in the text and in Figure 3 (new Figure 5).

6. Given the apparent impossibility of finding a model that is chemically satisfactory, the uncertainty about whether the molecular conformations are eclipsed or staggered, and the calculated H -atom positions I consider the detailed interpretation of the structures starting

with Fig. 3 on pg. 9 unjustified. (The question of whether or not anagostic interactions are strong enough to account for a phase transition is not important to this conclusion.)

Authors respond: This is the clue of our paper and we agree that we failed to clearly explain it! This is the main point – providing the understanding of the mechanism governing the conformational polymorphism of prototypic metallocenes and the correlations between their transformations, hence we have tried to better elaborate on it in the revised version and added a new figure (Figure 4) for this purpose. We admit that we have not explained the significance of CH---M interactions sufficiently well.

As explained above in Point 5, the model built of equal populations of the staggered and eclipsed conformers is well grounded on our present work, together with the previous inelastic neutron experiments and Raman spectra (measured by other teams) – the references are given in our paper [11,16,20], while the contribution of anagostic CH···*M* bonds for stabilizing the eclipse conformation was proven without doubt in our recent paper reveling new high-pressure phase of osmocene and including theoretical computations of the charge distribution in $FeCp_2$, $RuCp_2$ and $OsCp_2$.[2] The computations clearly show that the magnitude of electronegative potential around the metal site grows in the sequence $FeCp_2$<$RuCp_2$<$OsCp_2$, which make the H-acceptor capabilities growing in the same sequence: the lowest for $FeCp_2$ and the largest for $OsCp_2$. Most importantly, the computations showed that the magnitudes of the negative electrostatic potential on the molecular surface about the metal atoms are significantly larger for the eclipsed conformers and that the formation of anagostic bonds halts the rotations of the rings due to the steric hindrances intensified for the staggered conformers. We have plotted these electrostaticpotential magnitudes to visualize this sequence. We also have plotted the intermolecular H···H distances, which result in steric hindrances for assuming the staggered conformers.

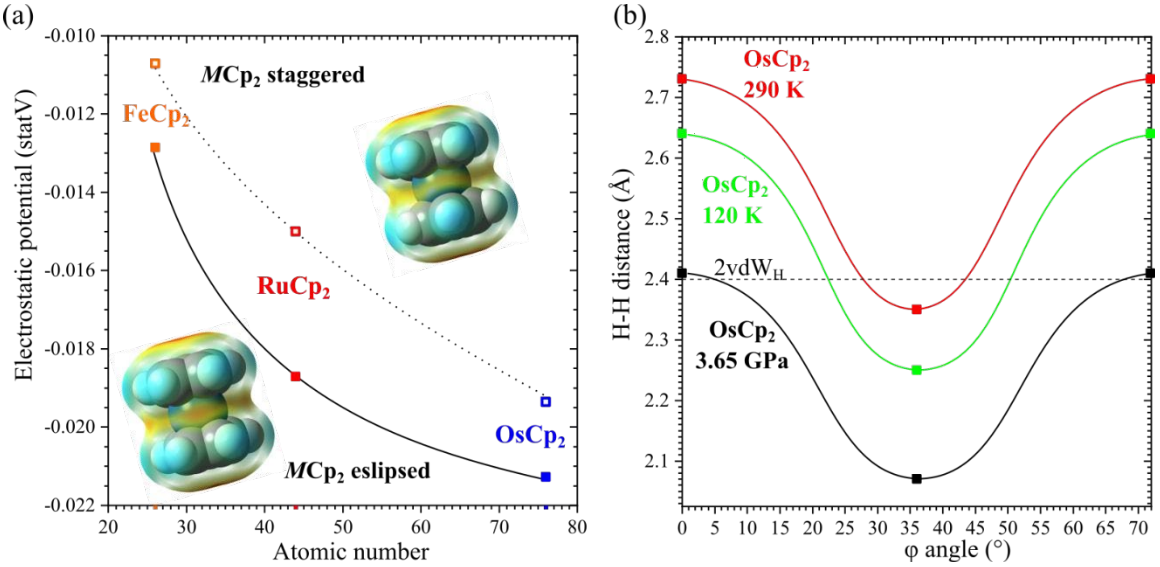

Figure. (a) Electrostatic potential on the molecular surface about the metal in ferrocene, ruthenocene and osmocene molecules; the insets show the electrostatic potential calculated for the molecule of osmocene in the staggered and eclipsed conformations.(b) Intermolecular H···H distances of the H atom involved in the anagostic bond to the closest H atom of the Cp ring in the experimental osmocene structures (eclipsed) and calculated for the other conformers.

7. The experimental work reported in this manuscript is important and should be published after revision (see comments above and below), but the experimental work does not support some of the interpretation presented and some of the conclusions drawn (such as the importance of anagostic interactions).

Authors respond: We hope that the arguments documented listed above in Point 6 with the consistent theoretical computations and experimental results are convincing that the anagostic bonds are important – we have improved their description and the explanation of their effects (see Point 6). Moreover, we stress that the strength of anagostic bonds, expressed in their shortness and the H-acceptor capabilities of the metal sites, well correlate with the transition temperatures destabilizing the Cp rings in ferrocene, ruthenocene and osmocene (Figure 6 – **new** number)

Less important points:

8. In the TOC graphic the disorder is shown for only two of the four rings. The metal atom lies on the origin, where the site symmetry is mmm. All four rings are therefore disordered.

Authors respond: We have improved the TOC by indicating the disordered sites of the rings superimposed with two carousel arms. We believe that it makes the 4-site disorder more apparent.

9. In Fig. S3 five points are shown for the Pnma Os structure but only three cifs are provided for that structure.

Authors respond: α-Osmocene was also measured at 350 K and 400 K, but the measurements were conducted on diffractometer equipped with CuK_{α} anode X-ray tube. This radiation is strongly absorbed in $OsCp_2$, so strongly that the absorption corrections were ineffective. Therefore we decided not to attach these structures to the manuscript, even though they were solved with R1 factors equal: 8.77 % and 10.63 %. The γ phases of ruthenocene at 400 K and osmocene at 427 K were measured at a diffractometer with MoK_{α} radiation.

10. On fig 6 it says "Above the transition temperatures the *c* parameters display a strong negative thermal expansion, also observed for parameters *a* and *b* of ruthenocene and parameter *b* of osmocene." but I cannot find data for the change with T of the cell constants of the Fmmm structures.

Authors respond: Thank you for this comment – indeed some of the measurements by powder XRD and by single-crystal XRD using CuKα radiation were not given in the manuscript. To better document the thermal changes of the crystals at the phase transitions, we have remeasured the thermal expansion of the ruthenocene crystal on one diffractometer and we have included the new results in Figure 2. The old and new results are consistent and show the negative thermal expansion of the ruthenocene crystals above the phase transition. We have completed all the unitcell measurements in the Supplementary Information Tables S2 and S3. We have also added the reference to these tables in the text.

11. In Table S1 should the "A" in the second row, second column be changed to an alpha?

Authors respond: We apologize for this mistake! Thank you for noticing it – it has been corrected in the revised version.

12. In the cif the chemical name is wrong in the two blocks containing information about the Ru Fmmm structure.

Authors respond: We apologize for this mistake! Thank You for noticing it – it has been corrected in the revised version of CIF files and in CCD database.

13. Why were 36 restraints needed in the refinement of the Os Pnma structure at 120 K but not at 200 K?

Authors respond: We have corrected the model and eliminated unnecessary restrains. However, at 120 K the ADP parameters in two of the C atoms need to be restrained with ISOR 0.01 while the quality of the 200 K data is better and it did not require this restrain.

14. Why do the numbers of variables for the three Fmmm structures (50, 47, 58) differ?

Authors respond: We are grateful for this comment. This different number of parameters was the result of differently modeling the H atoms in the structure. Thanks to your suggestion we have reanalyzed our γ models and now we apply the same model for all structures. Now there are 47 refined parameters for γ-ruthenocene and 48 for γ-osmocene.

Ru, Pnma in order of increasing temperature

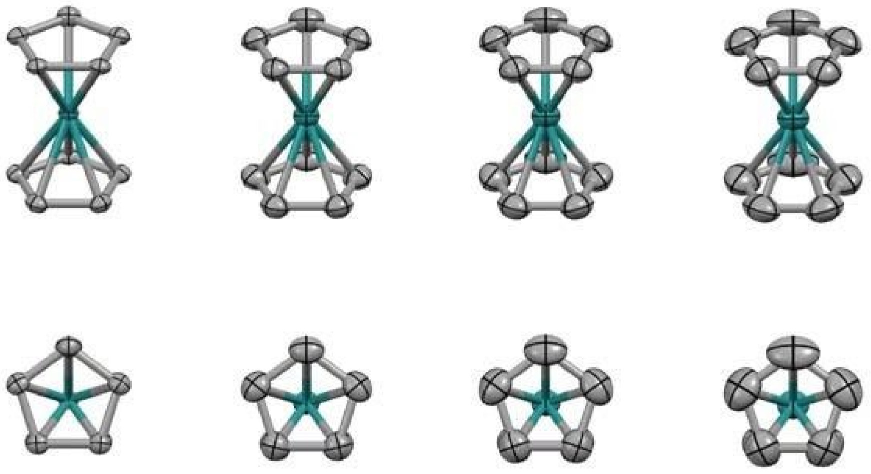

Os, Pnma in orderof increasing temperature

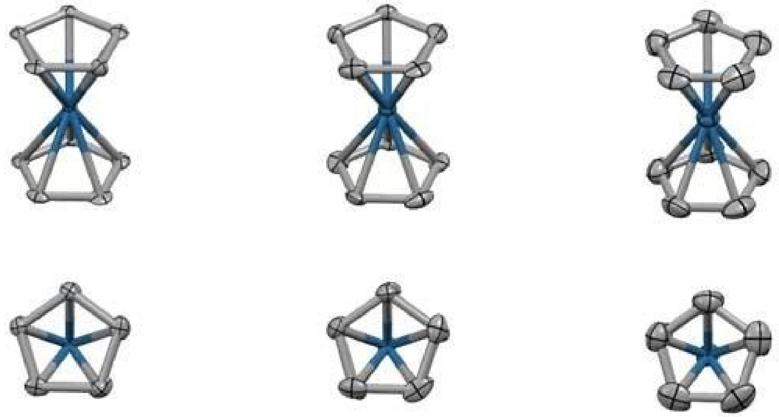

Ru, Fmmm at 393 K (atoms for one molecule only)

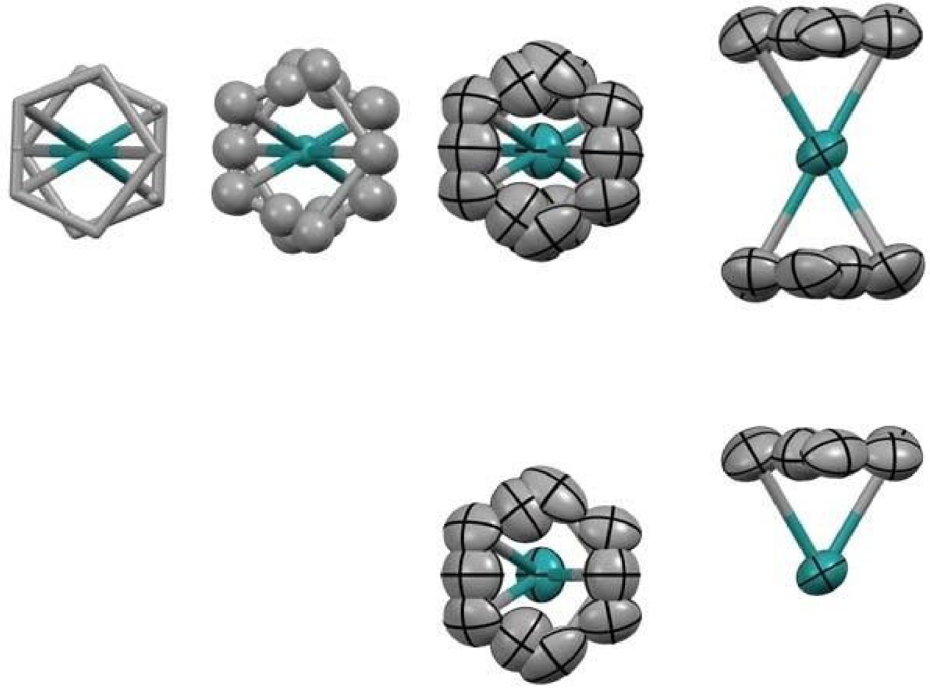

(1) Sheldrick, G. M. Acta Crystallogr. 2008, A64. pp 112–122.

(2) Moszczyńska, I.; Gulaczyk, I.; Katrusiak, A. Giant Deformation between Osmocene Phases Induced by Anagostic Bonds Promoted under High Pressure. *J. Phys. Chem. C* **2023**, *127* (38), 19250–19257. https://doi.org/10.1021/acs.jpcc.3c04579.

jz-2025-00686a.R2

Name: Peer Review Information for "New Osmocene and Ruthenocene Phases Reveal the Common Conformational Behavior Regulated by Anagostic Bonds in Prototypical Metallocenes"

Second Round of Reviewer Comments

Reviewer: 2

Comments to the Author

Review of jz-2025-00686a.R1

I have read the authors' responses and the revised files carefully. I used Acrobat to compare the original and revised manuscripts and looked carefully at the comparison document. I conclude that while the authors have made changes in response to many of my comments they have made no significant change to address my two most important comments, which concerned: (1) their claim of knowing the correlations between the disordered positions of the rings

(2) their claim that specific molecular conformations can be identified even though the electron density in the rings is approximately continuous.

In the authors' responses there are the sentences

"However, the inelastic neutron scattering, Raman spectra and the computational analysis show that the rotation of the "rigid" molecule by 36° are unlikely – practically impossible; these studies indicate that the rings rotate separately, and such a rotation of one ring has to involve the changes between the staggered and eclipsed conformers. We agree that this conclusion cannot be deduced from the X-ray diƯraction experiment alone. We have improved this explanation in the text."

I searched the revised manuscript for the words "neutron", "inelastic", and "Raman" but found no statement that was not already in the original manuscript. The word "Raman" appears in two references, but their titles indicate that the metals in the compounds studied were Fe and Ni only.

Another problem with the analysis of the phase transition is that identification of agostic and anagostic interactions depends on the positions of H atoms while in this study the only information about the positions of the H atoms comes from assuming standard bond lengths and angles in the Cp rings.

There are other problems, of which I will only mention a few.

Re

“This exceptional stability of ruthenocene (RuCp2)22,23 and osmocene (OsCp2)4,24 in the eclipsed conformation was puzzling, when taking into account the nearly identical molecular dimensions as those of ferrocene, the same configuration of 18 valence electrons, the similar potential energy favoring the eclipsed conformers(10–12).”

None of those references considers the Os molecule and only #10 considers the Ru molecule, for which the authors of #10 said they were unable to determine a barrier to rotation. The statement “nearly identical molecular dimensions as those of ferrocene” is just not true except in a very general sense. The M-C bond lengths do increase with the atomic number of M.

I continue to believe that the manuscript reports important experimental work but that the conclusions drawn about the mechanism of the phase transition are not justified by the experimental results.

Author's Response to Peer Review Comments:

Ref.: jz-2025-00686a.R1

Dear Editor,

Thank you for your letter of 5 May, 2025 and for the second round of Referee’s comments. We are very grateful to the referee for indicating our mistakes and for these insightful considerations regarding the disorder. We have accordingly corrected the paper. We have to say that the distinction between the hindered and free rotations of the rings are very fundamental and at the same time very refined, and perhaps the definite answer will have to wait for applying methods more appropriate for this purpose, i.e. still more infrared, Raman and quasielastic and inelastic neutron scattering – all these studies performed so far were for the temperature range up to 300 K, except for one study on nickelocene at 350 K, which of course is not relevant for the new phases gamma of ruthenocene and osmocene.

We are submitting the revised versions of our manuscript and its Supporting Information. Along with the manuscript and SI files, we are submitting their versions with all the changes highlighted: to facilitate the reviewing of changes, in the revised version we have kept the highlights of the first revision, and the second revisions are highlighted blue. The submission is accompanied with the response to all referees’ comments, enclosed here below.

With best regards,

Prof. Andrzej Katrusiak
Wydział Chemii UAM
Collegium Chemicum, Blok A, Biuro
1.19 Uniwersytet im. Adama
Mickiewicza ul. Uniwersytetu
Poznańskiego 8 61-614 Poznań
URL hpc.amu.edu.pl tel.:
+48 618291590 fax: +48
618291555 E-mail:
katran@amu.edu.pl

Responses to Referee's review

Review of jz-2025-00686a.R1

I have read the authors' responses and the revised files carefully. I used Acrobat to compare the original and revised manuscripts and looked carefully at the comparison document. I conclude that while the authors have made changes in response to many of my comments they have made no significant change to address my two most important comments, which concerned: (1) their claim of knowing the correlations between the disordered positions of the rings (2) their claim that specific molecular conformations can be identified even though the electron density in the rings is approximately continuous.

Authors response: *We are grateful for these insightful comments. Our knowledge about (1) the correlations between the disordered positions of the rings; and (2) the conformations; come from the combination of calorimetric and diffraction measurements, as explained below. We have tried to better present these aspects in the revised paper, including new Figures 5, S8 and S9.*

In the authors' responses there are the sentences

"However, the inelastic neutron scattering, Raman spectra and the computational analysis show that the rotation of the "rigid" molecule by 36° are unlikely – practically impossible; these studies indicate that the rings rotate separately, and such a rotation of one ring has to involve the changes between the staggered and eclipsed conformers. We agree that this conclusion cannot be deduced from the X-ray diffraction experiment alone. We have improved this explanation in the text."

I searched the revised manuscript for the words "neutron", "inelastic", and "Raman" but found no statement that was not already in the original manuscript. The word "Raman" appears in two references, but their titles indicate that the metals in the compounds studied were Fe and Ni only.

Authors response: *We fully agree with this comment – this is our mistake resulting from our fault due to a confusion caused by our recent publication on ferrocene phases, where these*

references were included. These “inelastic” papers should have been included in our present submission about ruthenocene and osmocene, too, which we have done now. The main point of these papers is the distinction of the ‘rigid’ and ‘soft’ models of the prototypic metallocenes. These ”inelastic” papers all confirm that the ‘soft’ behavior – i.e. the rotations of one Cp ring with respect to the other – is relevant. Unfortunately, to our knowledge there have been no such studies for high temperatures, even above 300 K, except one at 350 K for nickelocene. We hope that our paper will encourage such studies in the future. The added references are:

Appel, M.; Frick, B.; Spehr, T. L.; Stühn, B. Molecular Ring Rotation in Solid Ferrocene Revisited. J. Chem. Phys. 2015, 142. DOI: 10.1063/1.4915067.

Bodenheimer, J.S., Low, W. A vibrational study of ferrocene and ruthenocene (1973). Spectrochimica Acta Part A: Molecular Spectroscopy, 29 (9), pp. 1733-1743. doi: 10.1016/0584-8539(73)80125-4

Boeyens, J.C.A.; Levendis, D.C. Force field study of ring orientation in metallocenes. *S. Afr. J. Chem.* 1982, 35, 144-152. DOI ??????????

Braga, D. Dynamical Processes in Crystalline Organometallic Complexes. Chem. Rev. 1982, 92, 633-665. DOI 0009-2665/92/0792-0633

Ferreira da Cunha, T.; D. Calderini, D. Skouteris. Analysis of Partition Functions for Metallocenes: Ferrocene, Ruthenocene, and Osmocene. J. Phys. Chem. A 2016, 120, 27, 5282–5287. DOI: 10.1021/acs.jpca.6b01280

Gardner, A.B., Howard, S., Waddington, T.C., Richardson, R.M., Tomkinson, J. The dynamics of ring rotation in ferrocene, nickelocene and ruthenocene by incoherent quadi-elastic neutron scattering. *Chemical Physics* 1981, 57, 453-460. doi: 10.1016/0301-0104(81)80224-8

Kearley, G.J. A review of the analysis of molecular vibrations using INS. *Nuclear Inst. and Methods in Physics Research, A*, (1995) 354 (1), pp. 53-58. DOI 10.1016/0168-9002(94)00936-8

E. Kemner; I. M. de Schepper; G. J. Kearley; U. A. Jayasooriya. The vibrational spectrum of solid ferrocene by inelastic neutron scattering. J. Chem. Phys. 2000, 112, 10926 - 1092922. DOI 10.1063/1.481731

Page M. I. The energetics of neighbouring group participation. Chem. Soc. Rev. 1973, 2, 295-323. DOI 10.1039/cs9730200295

Parker, S.F.; Butler, I.R. Synthesis, computational studies, inelastic neutron scattering, infrared and Raman spectroscopy of ruthenocene. European Journal of Inorganic Chemistry, vol. 2019, no. 8 (2018): 1142-1146. DOI: 10.1002/ejic.201800914

Another problem with the analysis of the phase transition is that identification of agostic and anagostic interactions depends on the positions of H atoms while in this study the only

information about the positions of the H atoms comes from assuming standard bond lengths and angles in the Cp rings.

Authors respond: *The positions of cyclopentadienyl H-atoms fully depend on the ring position – perhaps the single-crystal neutron diffraction could yield comparatively accurate H positions as those derived from the Cp-ring geometry. Thus, the precise assignment of the type of interactions based on the H-atom position calculated from the Cp ring geometry is presently the state of the art. Let me add that at about 400 K, for a weakly interacting molecular crystal with flexible molecules, even the feasibility of singlecrystal neutron diffraction or the non-spherical atomic form factors refinements are questionable. The Hatoms are unequivocally located from the positions of their carriers and can be used for identifying the CH---M interactions, in this case anagostic bonds (agostic bonds are excluded due to the C-H---M angle far beyond the limiting opening of 140° and the C---M distance significantly too long). Another point is that the centers of the neighboring molecules shifted away, consistently with the CH---M bonds breaking.*

There are other problems, of which I will only mention a few.

Re

"This exceptional stability of ruthenocene (RuCp2)22,23 and osmocene (OsCp2)4,24 in the eclipsed conformation was puzzling, when taking into account the nearly identical molecular dimensions as those of ferrocene, the same configuration of 18 valence electrons, the similar potential energy favoring the eclipsed conformers(10–12)."

None of those references considers the Os molecule and only #10 considers the Ru molecule, for which the authors of #10 said they were unable to determine a barrier to rotation. The statement "nearly identical molecular dimensions as those of ferrocene" is just not true except in a very general sense. The M-C bond lengths do increase with the atomic number of M.

Authors respond: *We have added more references for the ruthenocene and osmocene molecules/crystals. We agree that there are some differences in the bond length to the M atom, and we have reformulated the sentence not to leave any doubt about it.*

I continue to believe that the manuscript reports important experimental work but that the conclusions drawn about the mechanism of the phase transition are not justified by the experimental results.

Authors reply*: Thank you for this positive assessment of our results and for the scientific discussion indicating the interesting aspects of our study. The dynamic properties of the metallocenes have been additionally indicated in the abstract and conclusions.*
